# Seed germination, morphology and fruit phenology insight of *Cylicomorpha solmsii (Urb.) Urb*: a step towards sustainable restoration planning

**DOI:** 10.1038/s41598-024-66018-9

**Published:** 2024-07-23

**Authors:** Raissa Fon Na-ah, Nadine Ndabeh Ngwa, Liliane Ngoune Tandzi, Eric Ngansop Tchatchouang, Dessireé P. Zerpa-Catanho, Emmanuel Youmbi, Libert Brice Tonfack

**Affiliations:** 1 Department of Plant Biology, Faculty of Science, University of Yaounde I, P.O. Box 812, Yaounde, Cameroon; 2 Department of Plant Biology, University of Illinois at Urbana-Champaign, Urbana, IL USA; 3 College of Technology, University of Bamenda, P.O. Box 39, Bambili, Cameroon; 4 Cameroon National Herbarium, P.O. Box 1601, Yaounde, Cameroon; 5 Institute of Sustainability, Energy and Environment, University of Illinois at Urbana-Champaign, Urbana, IL 61801 USA

**Keywords:** *C. solmsii*, Seed germination, Morphology, Fruit phenology, Light, Soil, Plant regeneration, Plant domestication, Conservation biology

## Abstract

*Cylicomorpha solmsii (Urb.) Urb* (Caricaceae) is a wild relative of domesticated *Carica papaya* native to the humid tropical forest of Cameroon. *C. solmsii* is becoming extinct due to rapid urbanization of its habitat. There is currently no restoration planning, no available data on seed germination, details on morphological description and fruit phenology. We investigated the effects of light and soil on seed germination, updated its morphological description and provided cues of its fruit phenology. In two series of experiments, a germination test was first conducted under light and dark conditions with three seed pre-treatments (scarification, drying and cold). Secondly, pre-treated seeds were sown in native soils of *C. solmsii* habitat collected at Eloumden I and II, two *ex-situ* and mixtures soil with sand. Qualitative and quantitative data were collected on different part of the plant and analyzed using R package version 4.3.2. Our findings showed that *C. solmsii* seeds can germinate only under light*.* The seeds manifested a physiological embryonic dormancy. The native soils showed the highest germination percentage and seedling establishment. The dioicy of *C. solmsii* was clearly described with incomplete staminate and pistillate unisexual flower whorls. *C. solmsii* was observed to produce fruits throughout the year at varying intensity. This information is a vital cue to species restoration and policy makers towards *C. solmsii* conservation*.*

## Introduction

*Cylicomorpha solmsii (Urb.) Urb* and *Cylicomorpha parviflora Urb.* are the sole endemic tropical African wild relatives of the 38 known Caricaceae species; commonly known as the stem core ancestral progenitors of the other five Neotropical genera, including *Jarilla, Horovitzia, Vasconcella and Carica*^1–5^. From time immemorial, *C. solmsii* has been employed in traditional medicine to treat food poisoning, malaria, chest pain in children and sexually transmissible infections such as gonorrhea^6^. Pouny et al.^7^ reported the first empirical research findings on *C. solmsii,* demonstrating a strong anti-cancerous activity of isolated quinolizidine alkaloids known as cylicomorphins against an HCT-116 cell line. Moreover, C. *solmsii* has been forecasted as a potent genetic pool that could be harnessed in *Carica papaya* breeding program as a sister out-group species to improve its performance in agriculture, food, energy, pharmaceutical industries and biomaterial production^9^. Unfortunately, apart from the laid down morphological description of this potent wild species in 1893^1^, its little known biological information is widely scattered in very few outdated publications with the emphasized need for warranted research^1–4,7–12^. Exacerbated by the fact that *C. solmsii* is a reported IUCN (International Union for Conservation of Nature) red list species with a severe decline of its wild population in the last decades (for agriculture purposes, wood exploitation, urbanization), and its emergence in new geographical ranges^11,12^; it is therefore crucial to develop strategies that will contribute to the efficient domestication and conservation of the germplasm of this endangered species.

No scientific information has been documented on the seed germination of *C. solmsii*. So far, 50–90% of wild plant species have been reported to produce seeds that are dormant upon maturity^12^. This aforementioned phenomenon seems to be a norm within the Caricaceae, leading to their obligatory subjection to either a physical or chemical pre-sowing treatment conditions to alleviate dormancy for seed germination to be improved^13–15^. For instance in wild *C. papaya*, the seed germination rate is only 6% with its gelatinous sarcotesta been the major inhibitor. However, pretreatment practices such as the removal of its sarcotesta by washing, drying and cold scarification could significantly improve seed germination^13,16–20^. Most importantly, environmental variables such as light, soil physical and chemical composition, temperature, water, relative humidity have shown to be key determinants of seed germination and seedling establishment in most crop species, including *C. papaya* which have thoroughly been studied as a role model species within the Caricaceae^21–23^*.* Therefore, understanding the seed dormancy status of *C. solmsii* and its germination requirements will be informative cues for seed restoration experts/practitioners to understand the nature of the seeds and identify possible environmental triggers that could possibly be optimized to obtain maximum outcomes.

Meanwhile the seed germination data is crucial; however, other indispensable guided questions to restoration planners are: (1) what are the distinctive morphological features of *C. solmsii*? and (2) what calendar month of the year is best to harvest seeds for future research? Although the trend in modern day research is a customary rush into molecular studies, the role played by genes and proteins are better understood once a detailed description of a plant form has been made^24–27^. Consequently, it is important to revise or update the initial laid down of morphological description of *C. solmsii*^1^, which was classically based on the use of natural illustrative methods to morphologically characterize plants from pressed and dried herbarium specimens based on first-hand observations. Although, this technique was informative but it is fast losing its applications in recent years due to ambiguities in description and several missing gaps of many useful morphological descriptors or traits that are loss in the course of pressing and drying^24,26^. Photographic images and hypothetical digital tools have attracted interest as promising alternatives to the use of pressed and dried collections, and have been extensively used to describe and understand the morphology of many plant species including some genera of the Caricaceae such as *Carica*^28–30^, *Jacaratia*^31,32^, *Jarilla*^33–35^, *Horovitzia*^36^ and *Vasconcella*^5^. Thus, revising the morphological description of *C. solmsii* with recent illustrative photographic images constitutes a quintessential knowhow in understanding its biology for strategic conservation planning. The objectives of this study were to (1) investigate the effect of light and pre-sowing seed treatment conditions on the seed germination of *C. solmsii* (2) determine the suitable soil growth medium for *C. solmsii;* by assessing the effect of soil from its endemic habitat (Eloumden) and two agroecological ex-situ sites, including University of Yaounde I in Cameroon on seed germination and seedling establishment (3) characterize morphological features of wild individuals using standard morphological descriptors and recent photographic images (4) assess its various fruit phenological stages within a period of 2 years.

## Results

### Effect of growth media and seed pretreatment on the seed germination of *C. Solmsii*

**Figure 1:**
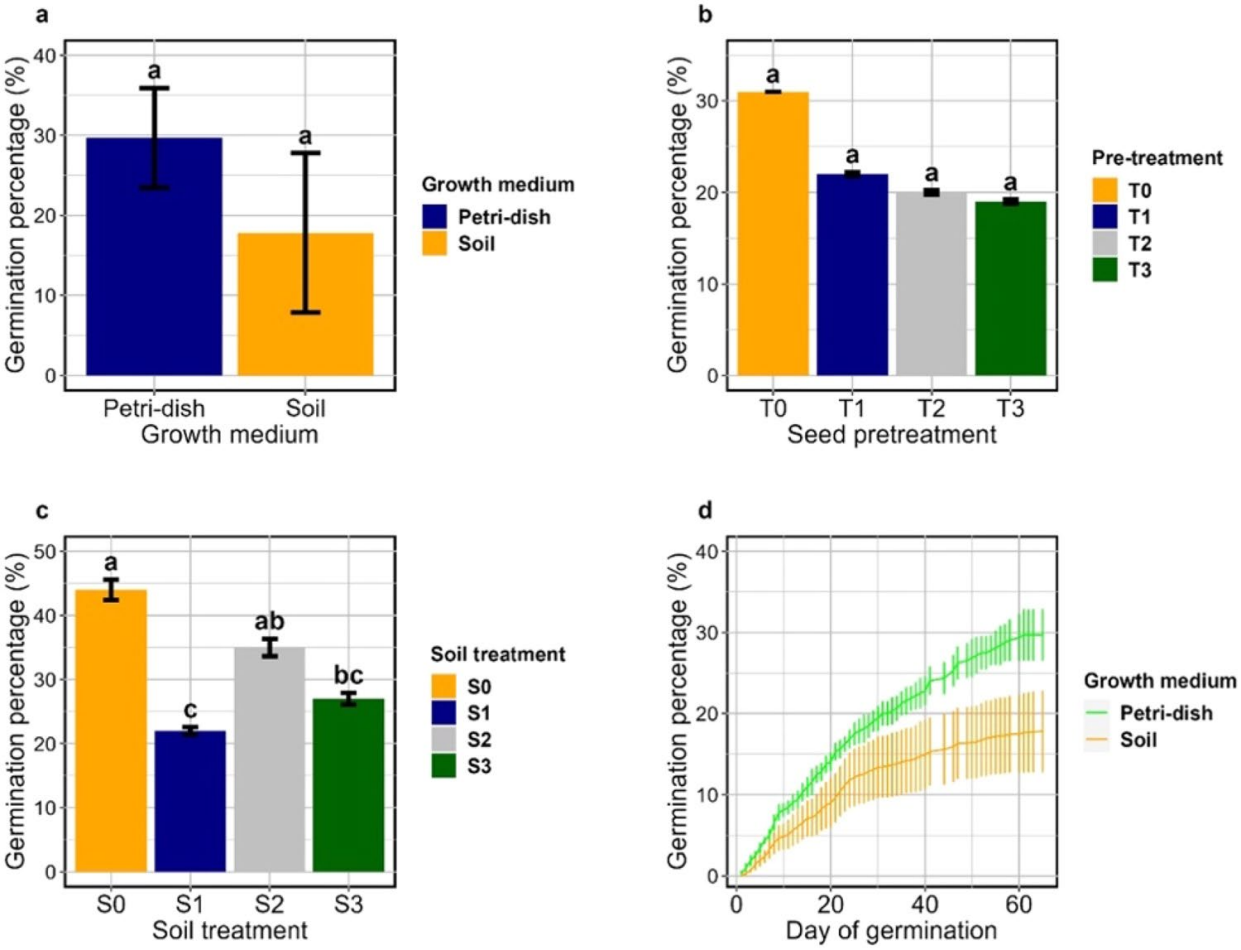
Effect of growth medium, seed soil treatment and time analysis graph on the germination percentage of *C. solmsii*. (**a**) Mean germination percentage on Petri-dishes and the soil. (**b**) Mean cumulative germination percentage for different seed pretreatments. (**c**) Mean cumulative germination percentage on different soil type. (**d**) Time analysis graph of the cumulative germination percentage from the start date of germination to the end on the soil and petri-dishes. Significant differences between the treatment groups are marked with small case letters.

Light strongly promoted the seed germination of *C. solmsii* with an observed percentage rate of 38.75% on Petri-dishes under light conditions, but no germination was observed in the dark. There was no significant difference (*P* = 0.089) between seed germination on Petri-dishes and soil, revealed by their mean comparison outcome (Fig. 1a, Table S3 and S4). Nonetheless, the highest germination percentage was obtained on the experimental setup in Petri-dishes (38.75%) compared to the soil with lowest values (7.25%) (Table S4 and S5). Analyzing germination within the seed treatment group and the soil group revealed that; (1) seed scarification pretreatment conditions had no significant effect (*P* = 0.67) on the seed germination of *C. solmsii* through the mean comparison of their cumulative germination percentage; although freshly harvested or unscarified seeds showed the highest values of 31% (Fig. 1b and Table S6). However, (2) soil type showed a marked influence (*P* = 0.0002) on seed germination rates with a decreasing order of magnitude from its parent soil through the soil, to no germination on the soil of Urbana-champaign. Cumulative germination percentage mean germination comparison results (Fig. 1c and Table S7) revealed no significant difference between the parent soil (44%) of El-I and El-II (S0, sandy-loam), and its corresponding pretreatment with sand (35%) in a 3:1 ratio (S2, sandy-loam + sand). However, the cumulative germination percentage was significantly lower (27%) on the parent soil of Yaounde 1 (S1, clay-loam) and its corresponding S3 (clay-loam + sand) treatment in a 3:1 ratio (22%), with no significant differences between the two-soil group. Germination began 21st day after sowing until the 65 day both on Petri-dishes and the soil, with no significant difference between the two groups when comparing the mean germination time (Fig. 1d; Table S8 and S9).

### Effect of soil on the seedling establishment of *C. Solmsii*

**Figure 2:**
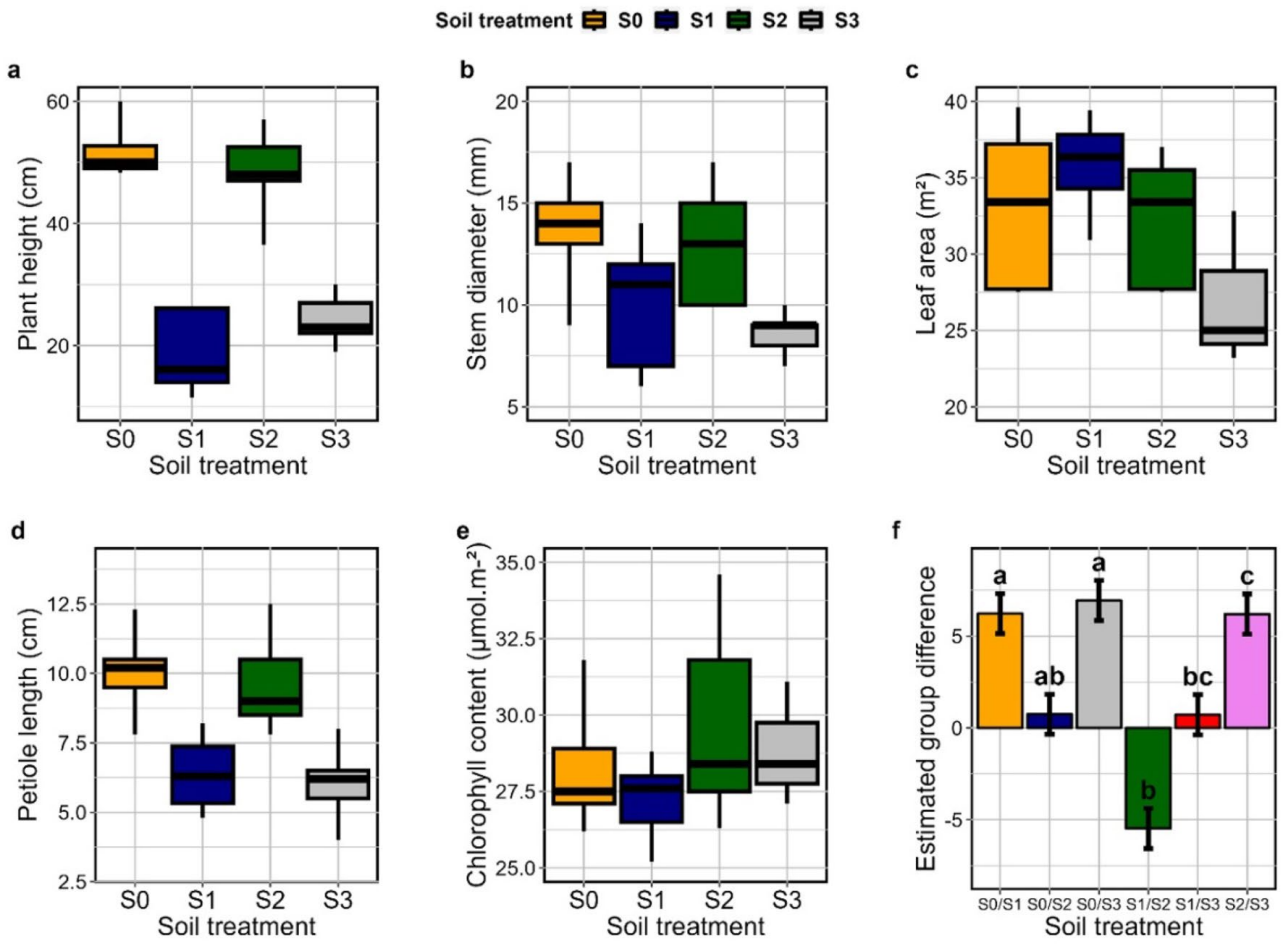
Effect of soil types different seedling growth parameters of *C. solmsii* 21 days after seed germination on: (**a**) seedling height (cm); (**b**) stem diameter (mm); (**c**) leaf area (m^−2^); (**d**) petiole length (cm); (**e**) chlorophyll content (μmol m^−2^); (**f**) Post-Hoc HSD Tukey’s test (*P* ≥ 0.05) with pairwise comparison of the mean in the different soil groups. Soil groups with the same lower-case letters do not differ significantly (S0/S2, S1/S3), whereas those groups with unique lower-case letters represent soil groups that differ significantly.

The different seedling growth parameters showed varying responses to soil treatment (Fig. 2a–e). There was no significant differences between all the soil treatment on seedling growth as shown on the pillai test (*P* = 0.084) (Table S10 and S11). Analysis of variance results on the effect of soil on each seedling growth parameter revealed that soil had strong influence on plant height (*P* = 1.783e^−07^), petiole length (*P* = 0.0016), a slightly lower influence on stem diameter (*P* = 0.03452), and did not affect leaf number (*P* = 0.48), chlorophyll content (*P* = 0.3487) and leaf area (*P* = 0.601) (Table SS12). Pos-Hoc mean comparison analysis revealed that the parent soil of Eloumden (S0), and its corresponding associated 3:1 sand treatment (S2) had the same influence (S0/S2) on seedling growth (Fig. 2f and Table S13). A similar trend of equal significance was observed with Yaounde1 soil (S1) and its associated sand mixture (S3) (*P* = 0.91). Modeling the effect of soil on the seedling establishments revealed varying influences, and the parent soil from Eloumden had a very strong significant influence on the seedling establishment of *C. solmsii* (*P* = 4.216e^−12^) compared to the other soil treatments (*P* = 0.085) (Table S14 and S15).

### Leaf morphology

**Figure 3:**
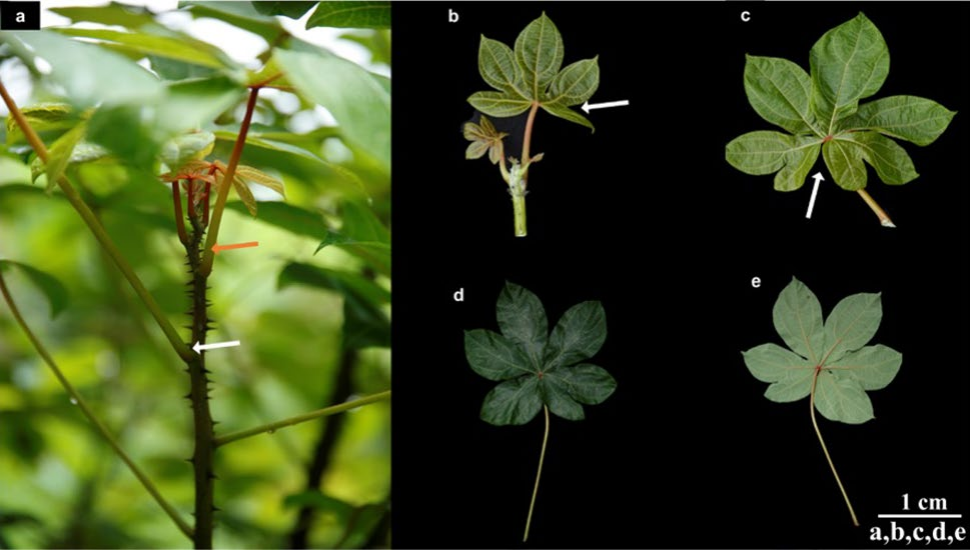
Structure of *C. solmsii* leaves. (**a**) Phyllotaxis. showing alternate leaf arrangement, the white arrow points to node without stipules (exstipulate) and the orange arrow point to petiole that joins the leaf to the stem; (**b**) leaf margins with white arrow pointing to an incised margin; (**c**) leaf base with the white arrow pointing a deep cordate notch; (**d**) deep green appearance of the upper leave surface; (**e**) pale green appearance of the lower leave surface.

The leaf of *C. solmsii* appears as a lateral appendage on the stem with a exstipulate leaf base or hypopodium (Fig. 3a). They are arranged on the stem in acropetal manner, with older leaves at the bottom and younger leaves at the top. The leaves are attached to the stem by a thin long flexible petiole (20–55 cm long; 4–8 mm in diameter,) inclined either horizontally or vertically upwards in alternating pattern. The color of the leaf petiole was observed to be highly variable, ranging from pale green at the leaf base to reddish green along its margins, and red at the region close to the junction near the leaf blade or lamina (Fig. 3a, Table S16). *C. solmsii* has a compound palmately lobed leave with a broad lamina or epipodium with a size range from; 6–12 mm in diameter and 15–27 m long (Fig. 3a-e, Table S2). The leaf margins are deeply incised with 3–5 irregularly shaped lobes (Fig. 3a-e, Table S16). The lamina surface was observed to be glabrous (smooth), with a deeply cordately widened notch base, which narrows elliptically at the middle of each leaflet and tails off acuminately at the apex (Fig. 3c). The leaf has prominent palmately-netted veins, with 3–4 primary veins, and numerous adjacent reticulate or net-like secondary veins (Fig. 3a and c). *C. solmsii* has a papyraceous texture and exudes a milky white latex when cut. The upper surface of the leaf lamina appears to vary with age: very young leaves appear to be red (Fig. 3d); middle age leaves, yellowish green (Fig. 3c) and matured leaves dark green (Fig. 3d). Whereas the lower surface of young leaves appeared indistinguishable from its lower surface at young stages but appeared to be pale green at maturity (Fig. 3e).

### Stem/trunk morphology

**Figure 4:**
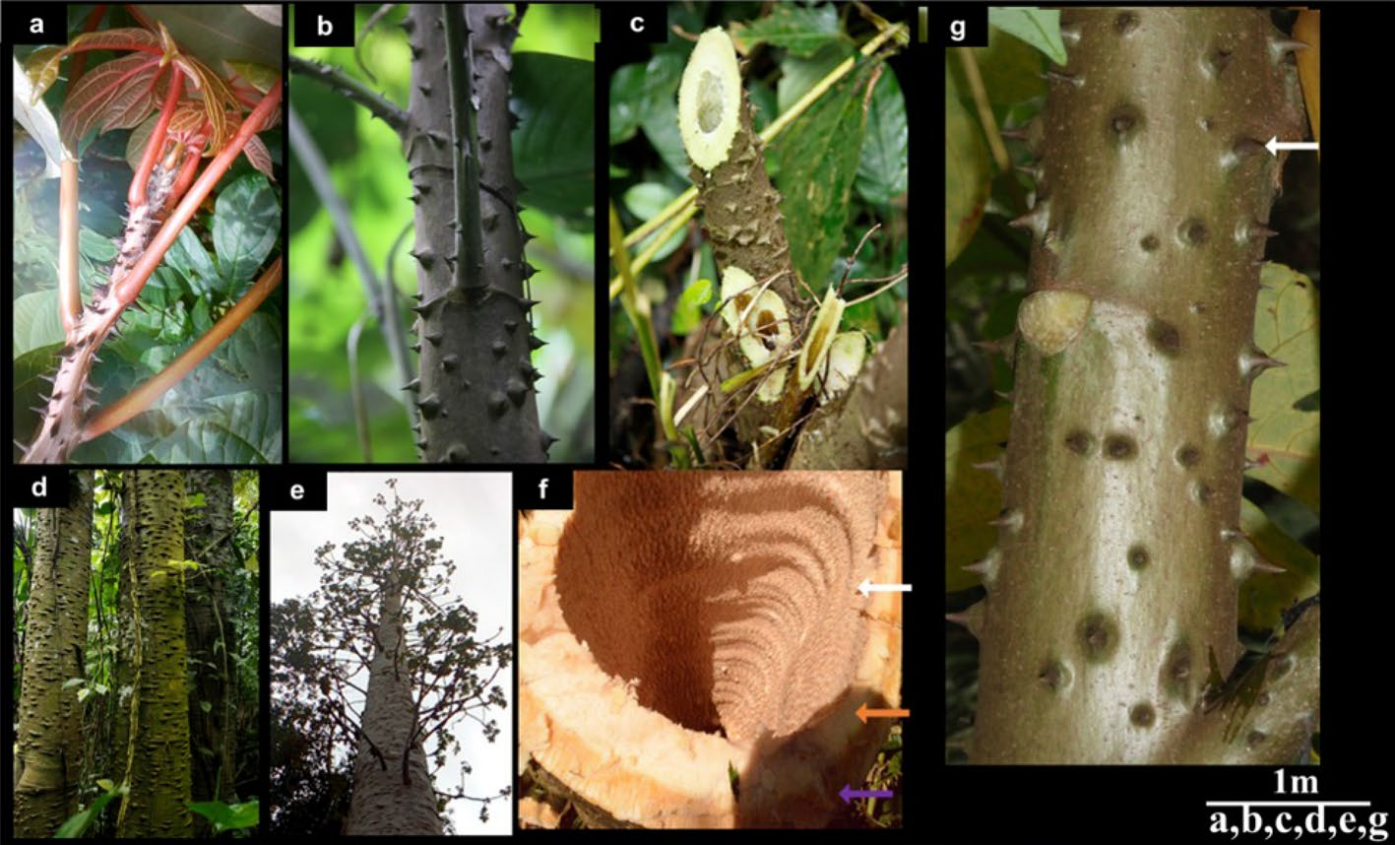
Structure of *C. solmsii* stem/trunk. (**a**) Young herbaceous stem at the apex; (**b**) single stem showing monopodial branching pattern; (**c**) cross section of a young stem, showing hollow pith cavity; (**d**) structure of the tree trunk; (**e**) semi-deciduous, perennial arborescent habit; (**f**) cross section of the woody trunk, the white arrow points to the heartwood with annual rings; the orange arrow points to the sapwood and the purple arrow points to the cambium; (**g**) Bark of the trunk with the white arrow pointing to sharp pointed prickles and white region scares of fallen leaves.

*C. solmsii* showed a shape gradient along its stem or trunk; apically the tree appeared herbaceous (Fig. 4a); towards the base, semi-woody (Fig. 4b,c); and basally, woody (Fig. 4b–g) marked by scars of fallen leaves (Fig. 4g). The above ground architecture was observed to be arborescent, with an unbranched hollow cylindrical trunk (Fig. 4d–f). *C. solmsii* exhibits a monopodial branching pattern, which is observed to grow indefinitely (Fig. 4e). The bark of the trunk was observed to be smoothly configured, conspicuously armed with prickles, emerging as defense outgrowth from the epidermis, and was borne throughout the bark (Fig. 4g). The prickle appeared to change shape pattern as very sharp pointed structures at the apex of the tree (Fig. 4a) to a conical spiny excrescence on the woody bark (Fig. 4d). The stem or trunk was observed to profusely produce a white, milky latex exudate when struck. The trunk color varied with structure, age, and season. Generally, the apex appeared red, then it changes to dark brown and then to grey towards the base (Table S16).

### Reproductive morphology of *C. solmsii*

*Cylicomorpha solmsii* was seen to be strictly dioecious, producing male (staminate) and female (pistillate) flowers on separate individuals. The reproductive structure appeared to distinctively differ from each other in terms of inflorescence, structure and flower arrangement (Table S17).

### Male inflorescence and structure of the staminate flower of *C. solmsii*

**Figure 5:**
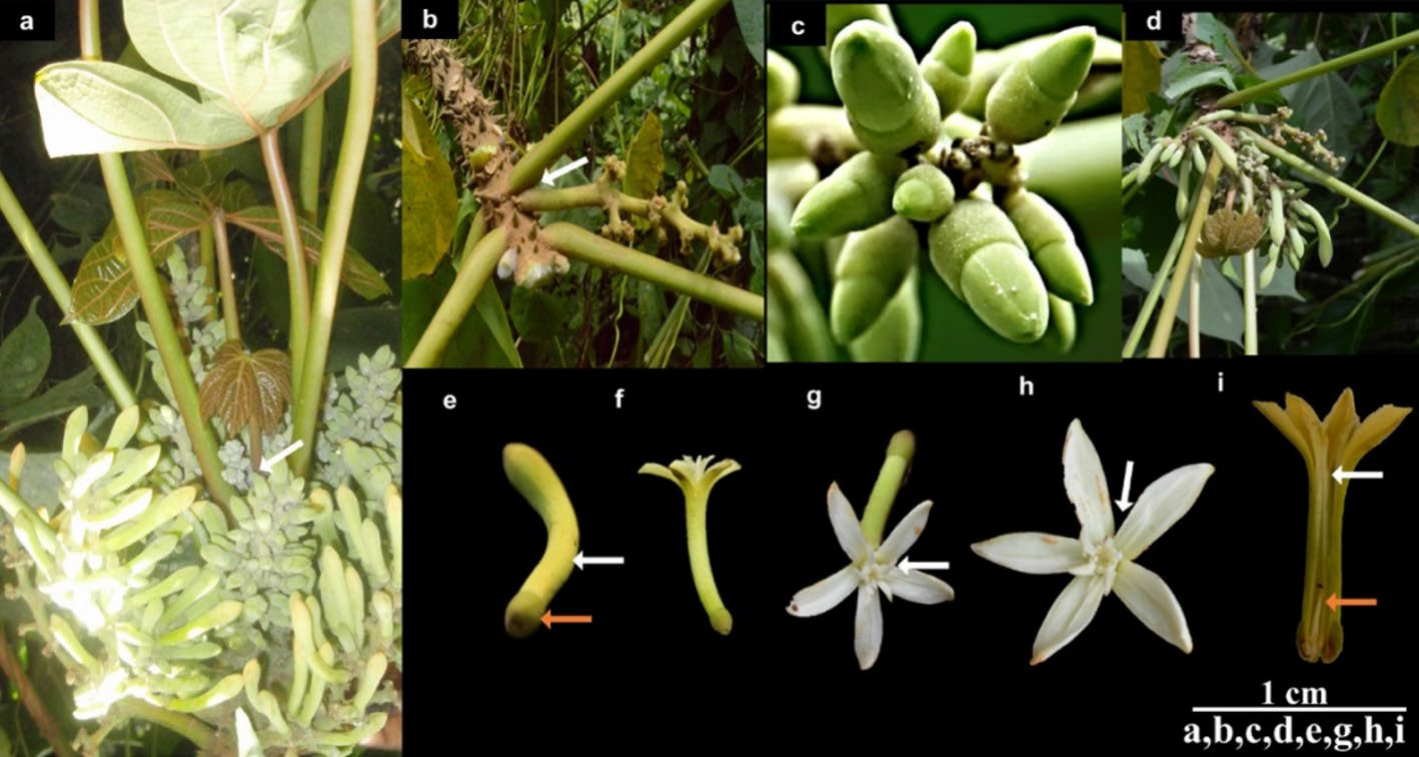
Inflorescence and flower morphology of *C. solmsii* stem/trunk. (**a**) Compound racemose. White arrow pointing to tip with young shoot showing continuous growth; (**b**) position of the peduncle at the axil of the leaf; (**c**) very young male stamen; (**d**) developing matured male stamen with inflorescence; (**e**) immature male flower with the white arrow point to the corolla and orange arrow pointing to the cupuliform-shaped calyx; (**f**) matured male flower with open five free petals; (**g**) stamens with two whorls of stamen, five in each whorl; (**h**) valvate-aestivation arrangement of the petals; (**h**) valvate-aestivation arrangement of the petals; (**i**) androecium attached to the inner wall of the corolla tube (epipetalous)-white arrow, and orange arrow pointing to a rudimentary pistil or pistiloid (non- functional ovary or vestigial ovary).

The staminate inflorescence was observed as compound racemose, positioned along the axil of the leaf (Fig. 5a,b). The tip of the main floral axis or penduncle showed an indeterminate growth (Fig. 5a) with cluster panicles arising laterally in alternating manner (Fig. 5b and d). Flowers were arranged acropetally; with younger ones at the tips and older ones at the base (Fig. 5c,d). The male perianth was described as hetero-chlamydeous with a distinct calyx and corolla arranged in two whorls around the stamen. The calyx was observed to be fused or reduced (gamosepalous) and had a cup-shaped or "cupuliform" structure. In immature staminate flowers, the corolla appeared fused; however, it opens at maturity into five free petals (polypetalous) having a salverform or trumpet shape structure (Fig. 5e,f). Petals showed a valvate-aestivation, arranged adjacently to each other in a spiral manner (Fig. 5g,h). The androecium or male reproductive organ had ten stamens arranged in two-whorls, with five in each whorl, which were all attached to the inner wall of the corolla tube (epipetalous) (Fig. 5g,h). Each stamen was made up of a filament, a dithecal anther, and a rudimentary pistil or pistiloid also known as a non- functional or vestigial ovary located at its base (Fig. 5i). The imperfect (unisexual) four incomplete cycly of the staminate flower is summarized by this floral diagram: [⊕ ♂ K _(1)_C_5_^⤺^A _(5) +(5)_]. Where; ⊕  = actinomorphic or radial symmetry, ♂ = male or staminate flower, K = corolla, C = calyx (C), A = androecium or stamens, ^⤺^ = androecium attachment.

### Structure of the pistillate flower of *C. Solmsii*

**Figure 6:**
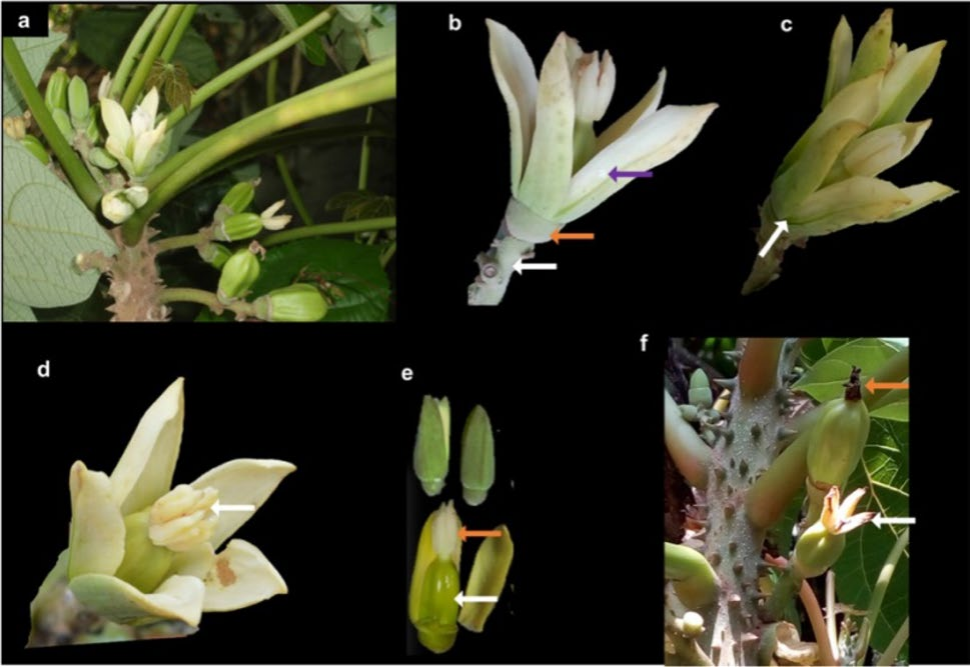
Structure of *C. solmsii* pistillate flower. (**a**) Position of flower inflorescence. (**b**) flower perianth. White arrow pointing to the flower pedicel; orange arrow cup shape calyx and the purple arrow pointing to the fleshy petals; (**c**) fleshy petals with white arrow pointing to its valvate aestivation; (**c**) the gynoecium, with white arrow showing the stigma. The gynoecium showing the ovary with a white arrow and stigma with the orange arrow (**f**) developing fruit from the ovary with deciduous stigma shown with the white arrow and persistent stigma on the developing fruit shown with the with the orange arrow.

The flower arises from the leave axillary near the apical region of the stem on several branching axes, bearing short pedicellate solitary flowers (Fig. 6a). The perianth was heterochlamydeous with a reduced "cupuliform" shaped sepal and five free fleshy petals, arranged adjacent to each other in a valvately aestivated manner (Fig. 6b,c). The gynoecium or pistil or carpel (female reproductive organ) was found only on female individuals. The gynoecium was observed to be made up of five feathery stigma which was directly connated to the ovary with no style. The ovary appeared to be superior and syncarpous, with five fused carpel or ovules in a parietal placentation. Also, the ovary was observed to have multiple planes of symmetry (actinomorphic symmetry) (Fig. 6e,f). The following floral diagram was used to summarize the female whorl of *C. solmsii* [⊕ ♀K _(1)_C_5_G_(5)_]. Where; ⊕  = actinomorphic or radial symmetry, ♀ = female or pistillate flower, K = corolla, C = calyx (C), G = pistil or gynoecium.

### Fruits, seeds, seedling morphology

**Figure 7:**
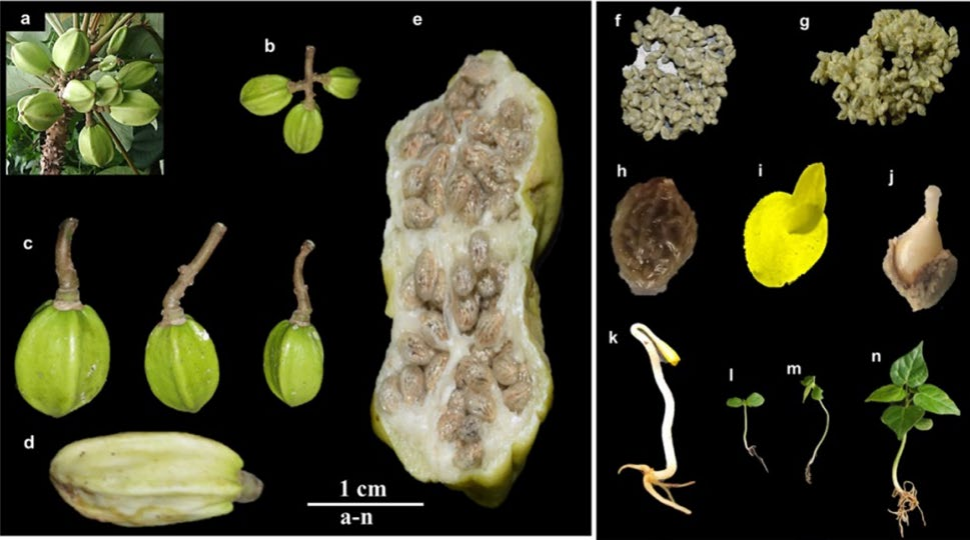
Fruit and seeds of *C. solmsii*. (**a**) Fruits on a single branch peduncle; (**b**,**c**) cluster and single fruits; (**d**) ripe fruits; (**e**) cross-section of the fruit with showing the septum; (**f**) immature seeds; (**g**) matured seeds; (**h**) a seed with gelatinous sarcotesta; (**i**,**j**) emerged radicle from a germinated seed; (**k**) germinated seeds with cotyledons; (**l**,**m**,**n**) 1, 2 and 4 weeks old of seedlings respectively.

The fruit of *C. solmsii* was described as; a simple fleshy, multiseeded berry (Fig. 7a and e) varying in size from 3.3 to 5.8 cm in length, and 26–36 mm in diameter (Table S18). Each fruit was attached to a long pale-brown peduncle with a length range of 4–8 cm, a diameter of 9–11.5 mm; and a weight range from 19.76–36.78 g. The fruit had a smooth skin texture with an ovate (ovoid) or elliptic (elongated) shape (Fig. 7b,c). The central cavity of the fruit is observed to be petitioned by a thin septum into a penta-locular chamber; with seeds arranged in five rows on the ovary wall in axile placentation (Fig. 7e). Immature fruits were observed to be lime-green in color (Fig. 7b,c), profusely producing a milky cream white latex when cut; whereas matured fruits tend yellowish green when they were ripe (Fig. 7d,e) and exudated transparent granular latex. The seeds of *C. solmsii* were relatively small, with various morphometric ranging parameters per seed; including length (0.5–0.7 cm), diameter (2–4 mm), number per fruit (14–318), fresh weight (0.23–0.59 g) and dry weight (0.001–0.04 g) (Fig. 7f-h, Table S19). At physiological maturity, the seeds were striped golden-brown; while immatured seeds were striped whitish-brown coated by a mucilaginous sarcotesta (Fig. 7f-h). Typically, matured seeds had large albuminous or endospermous embryo (Fig. 7i) and was observed to be dispersed principally by water (hydrochory) and animals (zoochory) [as observed during our phenological studies]. Seeds sprounted as a dicot at germination described as epigeous or phanerocotylar (Fig. 7j), with two cotyledonous leafs elevated above the ground (Fig. 7k). After the growth of the embryo, the radicle predominates and becomes the main taproot system, with numerous secondary roots or lateral roots (Fig. 7l-n). Once germination was successful, *C. solmsii* ensued a rapid indeterminate growth in a gregarious habit as observed in wild individuals (Table S19).

### Fruit phenological stages

**Figure 8:**
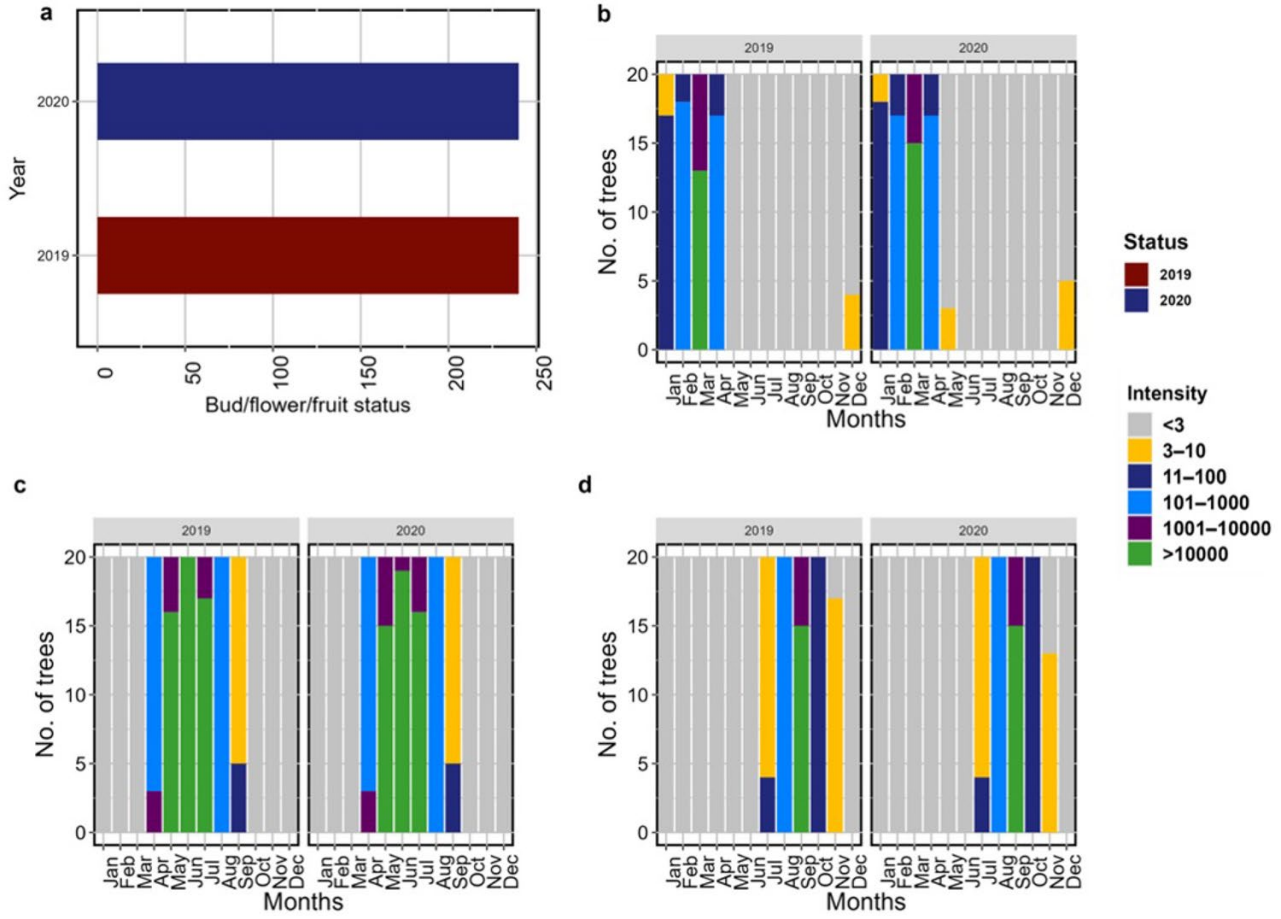
Fruit phenological stages of *C. solmsii*. (**a**) Bud, flower, fruit status; (**b**) flower bud intensity; (**c**) flower intensity; (**d**) fruit Intensity. Intensity; was an estimated count of a given phenophase (Bud, flower, fruit); represented by a number scale [< 3, 3–10, 11–100, 101–1000, 1001–10,000, > 10000] adopted from the USA National Phenology Network standardized protocol^37^.

For the twenty tree replicates for each of the phenological phases; including flower budding, flower and fruit, a total of 480 “Yes” phenological status observations were made for *C. solmsii* during our 2 years study period from January 2019–January 2021 (Fig. 8a). This investigation indicates that flower budding, flowering and fruiting of *C. solmsii* occurs throughout the year at different magnitudes quantified by their intensity (Fig. 8b–d). Flower buds were observed to gradually increase from December through April, peaking in March; followed by a gradual increase in flowering from April through July, with a peak in June. Fruiting on the other hand spanned June through October, peaking in September with the highest levels of ripe fruits. After a peak phenophase, there was a sharp decline to constant levels of 3% till the next season. Although, additional regression analysis is still required to fully explain this observation with climatic data (temperature and precipitation); our initial intensity analysis revealed significant variation of fruit phenological stages through the months of the year: flower buds (*χ*^*2*^ = 1295.8, *df* = 55, *P* = 2.2e-16), flower (*χ*^*2*^ = 1430.4, *df* = 55, *P* = 2.2e-16), fruit (*χ*^*2*^ = 1728.6, *df* = 55, *P* = 2.2e-16). However, no significant difference was observed between the two years: bud (*χ*^*2*^ = 0.58, *df* = 5, *P* = 0.98), flower (*χ*^*2*^ = 0.21, *df* = 5, *P* = 0.99), fruit (*χ*^*2*^ = 0.174, *df* = 5, *P* = 0.99).

## Discussion

In this study, we explored the seed germination of *Cylicomorpha solmsii*, investigated its morphological features as well as its fruits phenology; shedding light on its dormancy status, environmental triggers including light and soil that could interplay in the transition between domestication and its endemism. Firstly, regarding seed germination percentage it was generally low across all the treatments in our experimental setup with the highest record of 38.75%, intuitively suggesting seed dormancy. Kildisheva et al.^23^ highlighted that seed dormancy is the major challenge to the germination for 50–90% of wild species due to specific dormancy trait driven by factors, such as geographical occurrence, growth form and genetic makeup. Our initial investigation of the type of dormancy through mechanical scarification, with a control (unscarified seeds) and three treatments, revealed that the gelatinous sarcotesta of *C. solmsii* is not a possible inducer of seed dormancy or an impose limitation of germination, given the fact that there was no significant difference between the seed pretreatment and the control. Although, additional experiments with thermal and chemical scarification are needed to verify the dormancy status of *C. solmsii* However, these initial findings indicate that the dormancy of *C. solmsii* seed is most likely related to the physiological state. Contrary to our findings, the gelatinous sacrotesta of *C. papaya* has been shown to act as a major barrier to seed germination^18,38^. However, very low germination percentage observed in our findings seems to be a trend in some species of the Caricaceae, including *C. papaya* and *Jarilla chocola*^35,39,40^*.*

Light and soil were shown to be an environmental trigger that is indispensable for the germination of C. *solmsii*; as seed germinated only under light and not in the dark*.* This result is consistent with those observed in wild papaya seeds^41^ as well as in many other plant species where light has been reported as an important environmental factor that controls seed germination^42,43^. The profound effect of soil was revealed in the rhythmic change in results in both the germination test and seedling growth experiments; with the highest germination percentage records and better seedling performance on the parent soil from Eloumden. These results could be explained by the physical and chemical characteristics of soil samples from the different studied locations (Table S1). Soil from Eloumden I (76.44%) and Eloumden II (64.44%) had the highest percentage sand compared to the *ex-situ* soil from the University of Yaounde I (36.79%). It is highly probable that the embryo of *C. solmsii* is sensitive to soil oxygen availability, moisture content and drainage which are the driving forces in loosely aggregated sandy soils described as the most suitable for germination of tropical tree seeds due to their ability to permit the rapid exchange of respiratory gases between the embryo and the growth medium^44^. This observation corroborates with results in two tropical African trees, including *Acacia sieberana* and *A. drepanolobium*^45,46^*.* Despite the clogged texture of clay soil due to their compactness, seeds could still germinate, though with the lowest values (7.25%) and even more improved outcomes (12.50%) when mixed with sand in a ratio of 3:1 as opposed to silt soil with no germination. Though silt soils are principally coarser than clay containing soils^43^; however, the soil of UYI appeared to have a higher sand content and essential nutrient availability, including NO_3_^−^ (28.00 ppm), NH_4_^−^N (6.50 ppm) and Bay 1 phosphorus (6.50 ppm) (Table S1).

The compound palmately lobed leaf morphology observed in *C. solmsii* with four to five prominent main veins was a characteristic feature reported across the 40 species of the Caricaceae^4,5,47^. On the stem/truck of *C*. *solmsii*, prickles were identified to be densely distributed throughout the herbaceous apical stem and woody bark. This was congruent with finding reporting conical prickles in its East African sister relative, *Cylicomorpha parviflora*. However, inconsistent with previous inventory findings in *C. solmsii,* rather reporting the presence of sharp conical thorny spines on its woody bark^9,10,48^*.* The Prickles seem to act as mechanical defense structures in *C. solmsii*. Also, similar to the white latex phytochemical defense exudation produced among all members of the Caricaceae^4,26,49,50^, the stem/bark of *C. solmsii* profusely exude a white latex when struck.

The reproductive structure, including the staminate and pistillate flowers found on separate male and female individuals; robustly confirms previous reports identifying *C. solmsii* as strictly dioecious^1,9,10,48^. Moreover, the presence of a pistillode or a non-functional rudimentary ovary and style, a vestigial structure uniquely found in male individuals has being reported in *Carica papaya, Jacaratia mexicana* and across most of the reported Caricaceae, suggesting the pre-existence of hermaphrodism across most species within this family^4,28,51–53^*.* Intuitively, the vestigial ovary uniquely found in male individuals of *C. solmsii;* clearly suggests that a sex transition from a bisexual flower to an imperfect unisexual flower has once occurred in this species. Possibly, carpel development must have been suppressed in their hermaphroditic ancestors through several mechanisms; including genetic, epigenetic, and/or physiological factors earlier explained in *Carica papaya* as a model research species in this family^54–56^. The distinctive axile placentation identified in the female individuals of *C. solmsii* converse to the parietal placentation reported in *Carica papaya*^28,51^; insightfully could suggests that the unilocular placentation in *papaya* was probably derived from the fusion of the pentalocular axile placentation of *C. solmsii* during the course of their evolutionary history. Also, *C. solmsii* had a remarkable seed content (~ 202 seeds per fruit) with each seed enclosed by a gelatinous sarcotesta; closely similar to those in papaya^57,58^. This makes *C. solmsii* an additional promising reservoir for the exploration of papain production, previously reported as a valuable proteolytic enzyme extensively employed in the pharmaceutical, food and cosmetic industries extracted from papaya^58^. Flower buds, Flowers and fruits were observed on female tree throughout the twelve calendar months of the year, though with varying intensity as shown on Fig. 8**.** Fruit phenological stages of C. solmsii. (a) bud, flower, fruit status (b) flower bud intensity (c) flower intensity (d) fruit IntensityThis observation was similar to the fruit phenology of *Carica papaya*^59^.

This study is a promising beginning towards *Cylicomorpha solmsii* restoration and domestication; as it lays a foundation necessary to accurately predict the germination requirements in order to ensure its successful growth. Also, the initial laid-down morphological description of *C. solmsii* was updated with recent photographic images, and cues of fruit phenology was generated. Therefore, the baseline findings generated in this paper is crucial for restoration practitioners and policy makers towards a sustainable domestication and conservation of the germplasm of *C. solmsii* as an endangered IUCN plant species.

## Methods

### Study site and sample collection

The investigation geared towards the restoration planning of *C*. *solmsii* was conducted from January 2019–January 2021 at the Eloumden submontane forest (Eloumden I and Eloumden II) located at Mbankomo subdivision, Mefou and Akono division in the Center Region of Cameroon (Fig. S1). Eloumden is a humid tropical forest within the Equatorial rainforest in the Congo Basin^60^. The temperature and precipitation during our study period ranged from 20.57–23.6 °C and 0.85–180.44 mm respectively (downloaded from the Global Precipitation Climatology Centre (GPCC) database: GPCC gridded temperature and precipitation datasets). Sample material for seed germination, morphological characterization, phenological studies were collected from wild male and female individuals of *C. solmsii*. *C. solmsii* identified by botanists from the Department of Plant Biology, University of Yaounde 1 (Prof. Libert Brice Tonfack) and from the National Herbarium of Cameroon (M. Eric Ngansop Tchatchouang). Sample collection and research were done in compliance with the IUCN policy statement on research involving species at risk of extinction, the Convention on International Trade in Endangered Species (CITES) of Wild Fauna and Flora, and the Cameroon APA-law governing access to genetic resources, their derivatives, with traditional knowledge associated and the fair and equitable sharing of the benefits arising from their use. Representative voucher specimens of our collection of *C. solmsii* were deposited at the National Herbarium of Cameroon under the voucher number 6747 (Fig. S3 and S4).

### Physico-chemical analysis of soils used for seed germination testing

Soil for seed germination testing was randomly sampled from five locations at each sampling site, including (1) Eloumden I (EL I), (2) Eloumden II (EL II) at Eloumden; (3) University of Yaounde I (UY I) experimental farm located in the Mfoundi division in Cameroon (Table S2). Soil samples were collected at a depth of 20 cm from the topmost layer. The soil samples were allowed to air dry for 48 h, then ground with a 2 mm sieve. 3 g from each soil sample was packaged and submitted for physiochemical analysis at the Brook side soil analysis laboratory located at New Bremen, Ohio in USA (Table S1). Soil pH was determined using the lime requirement (LR) method^61^, and the soil organic matter was determined using the loss on ignition method^62^. Total nitrogen (N) was determined using the micro-Kjeldahl method; by the wet oxidation of organic matter using sulfuric acid (H_2_SO_4_). Exchangeable ions such as S, Ca, Mg, K, Na, B, Fe, Mn, Cu, Zn, Al, P and cation exchange capacity (CEC) were determined by the Mehlich–3 extractant method^63^; and Bray I Phosphorus was determined using the Bray & Kurtz-1 methods^64^.

### Seed pretreatment and germination testing experiment on Petri-Dishes

In mid-February 2021, prior to seed germination testing on the soil, a germination test was first conducted to evaluate two factors: Light/dark treatment, and pre-seed treatment by scarification on the seed germination of *C. solmsii.* The experiment was conducted under ambient conditions in the Plant Biotechnology laboratory at the University of Yaounde 1, Cameroon. Ripe fruits of *C. solmsii* used in this study, were randomly collected from ten female individuals from Eloumden submontane forest*.* Prior to sowing, seeds were extracted from the fruits and subjected to three scarification pre-treatment conditions, including (1) T1 (Scarified Fresh Seeds, SFS), fresh seeds were hand-scarified after removing the gelatinous sarcotesta; (2) (T2 (Dried Scarified seeds, DSS), seeds were allowed to air-dry under ambient conditions on kraft paper for one week; (3) T3 (Cold Scarified seeds, FSS), seeds were stored under cold conditions at 4 °C for two weeks; and (4) T0 (control), seeds were freshly harvested and sown with no pre-treatment. Seed were sown on 60 × 15 mm sterile disposable Petri dishes overlayed with two layers of Whatman filter paper (No. 1) moistened with 10 ml of distilled water. Just before sowing, each seed treatment lot was sterilized by washing in 1% dilute solution of sodium hypochlorite for 10 min, and then rinsed three times with sterile distilled water. For each pretreatment condition 10 seeds were sown in a Petri-dish with eight replications, making a total of 80 seeds per treatment. The experimental setup was tested under (1) light conditions, where seeds were exposed to natural photoperiod (12 h light:12 h dark), and (2) continuous darkness (24 h dark) where seeds were displayed in dark throughout the experimental period^65^. A seed was considered to have germinated after the emergence of its radicle. Germination was carefully monitored for 60 days. At the end of the experiment, the germination percentage (%) was calculated using the equation: G (%) = (A/B) × 100%; where A is the total number of seeds germinated at the end of the experiment and B is the total number of seeds sown^66^.

### Seed germination on different soil types and seedling growth

Following the response of *C. solmsii* seeds under the different scarification pretreatment conditions, the best performant seed pretreatment was used for germination experiment on soil. After analyzing the physiochemical properties of the soils from the four experimental sites, the soil samples were classified into three types; namely Sandy-loam, Clay-loam, Silty-clay loam according to Brady and Ray^67^, respectively representing soils from Eloumden I (El-I), Eloumden II (El-II) and University of Yaounde 1 (UY1)*.* The experiment was carried out at the agronomical research farm of the University of Yaounde I (latitude: 3° 84′, longitude: 11° 50′ and altitude: 710 m) under nursery conditions in May 2021. For the experimental setup at the University of Yaounde I, the soil from El-I and El-II (Sandy-loam) and UY 1 (Clay-loam) were used at four level treatment (two for each soil type), including (1) S0*,* Sandy-loam soil from El-I and El-II mixed in 1:1 ratio; (2) S1, Clay-loam soil from UY 1; (3) S2, Sandy-loam + Sand mixed in 3:1 ratio; (4) S3, Clay-loam + Sand mixed in 3:1 ratio. Twenty-five seeds were sown in each polyethylene bag at a depth of 2 cm, and laid in completely randomized block design with 16 replications for each treatment group. The seed germination percentage was determined 60 days from the planting date. Seedling growth parameters, including seedling height (SH); number of leaves (NL); leaf area (LA); stem diameter (SD); petiole length (PL) and chlorophyll content were measured 21 days after the emergence of the cotyledons.

### Statistical analysis

R version 4.3.2 software^68^ was used to perform statistical analysis. Germination indices, germination percentages, and cumulative germination percentage time series analysis were performed using the GerminaR package^69^. Statistical analysis was carried out using one-way analysis of variance (ANOVA), and mean comparisons was carried out using pairwise mean comparison method by Tukey’s HSD (*P* ≥ 0.05) test with the Emmeans package^70^. For seedling growth parameter analysis, multivariate analysis of variance (MANOVA) was used to assess the effect of soil treatment on seedling growth parameters using MASS package^71^. Prior to conducting MANOVA, independent assumption analysis was carried out through the Intraclass Correlation Coefficients (ICCs) using psych package^72^. Mean differences amongst the different soil groups were assessed through Pos-hoc analysis test using Tukey method^73^. To identify the potentially best soil treatment for the seed growth of *C. solmsii*, we used a linear model to evaluate the effect of soil independent variable *(X)* on the different seedling response variable *(Y)* as follows:

*Y* = *XB* + *E*, where *Y* = matrix of all the response variables (*Y1, Y2, Y3, Y4, Y5, Y6)*, respectively representing seedling height, number of leaves, leaf area, stem diameter, petiole length and chlorophyll content), *X* = the independent soil variable, *B* = the vectors of coefficients and *E* = errors of the residuals. Tests of statistical significance were carried out using Roy, Hotelling-Lawley and Pillai test.

### Morphological characterization of *C. solmsii*

Our investigation for the morphological characterization and fruit phenology of *C*. *solmsii* ran from January 2019 through January 2021 at Eloumden submontane forest (Fig. S1 and Fig. S2). For morphological characterization, ten trees, including five females and males were tagged from which photographic, tree parts and morphometric parameters were collected. Fresh leaves, stems as well as flower parts were collected for description. Photographic images of the various tree parts were captured using either a Nikon AF-S DX Zoom-Nikkor or an Olympus VG-160 digital camera. A slightly modified protocol was used to shape out leaf parts using a secateur, before they were mounted on a plant press paper; while the reproductive organs as well as fruits were labelled and stored in envelopes^74,75^. Specimens were later transported to the national herbarium of Yaounde in Cameroon for further description and morphometric measurement. Qualitative characters of the leaf, stem, flower, fruits and seeds were scored through reference comparison with standard morphological descriptor scoring scales, adopted from the International Board for Plant Genetic Resources^76^, and guided plant systematic resources^26,49,77,78^. Five replications for morphometric measurements from each tree were used for leaf (length and diameter); petiole (length and diameter); fruit (length, diameter, and weight); seed (length, diameter, weight and number).

### Fruit phenological stages of *C. solmsii*

Twenty female trees were tagged for observing the fruiting phenological stages. The USA National Phenology Network standardized protocol^37^ was adopted for scoring the status and intensity of flower budding, flowering, and fruiting of the selected female trees. Status observations were recorded as “Yes” indicating the presence or “No” indicating the absence of a phenophase stage. Because of the impose limitation due to the tree height (~ 40 m tall), the intensity of a phenological stage was quantified from six interval scales, including < 3, 3–10, 11–100, 101–1000, 1001–10,000, > 10,000 described by Denny et al.^37^. This scale reflected the phenophase abundance represented as percentages (< 5%, 5–24%, 25–49%, 50–74%, 75–95%, > 95%). A descriptive and chi-squared (*χ*^*2*^) statistical analysis with a level of significance at *P* ≥ 0.05 was performed in R version 4.3.2 software^68^, to evaluate differences within and between the two years**.**

### Supplementary Information

Supplementary Information.

## Data Availability

More data are furnished in the supplementary file. The raw datasets used and/or analysed during the current study are available from the corresponding author upon reasonable request and with the permission of the ministry of environment, protection of nature and sustainable development of the Republic of Cameroon. This work was done under the Cameroon APA-law governing access to genetic resources, to their derivatives with traditional knowledge associated and the fair and equitable sharing of the benefits arising from their use.
