# Supplementary material for: Seed germination, morphology and fruit phenology insight of Cylicomorpha solmsii (Urb.) Urb: a step towards sustainable restoration planning
Source: Sci Rep. 2024 Jul 23;14:16995. doi: 10.1038/s41598-024-66018-9 (PMC11266616; doi:10.1038/s41598-024-66018-9)
Supplement: Supplementary file 1 — Supplementary Information. [file 41598_2024_66018_MOESM1_ESM.docx]

**Seed germination, morphology and fruit phenology insight of *Cylicomorpha solmsii (Urb.) Urb*: A step towards sustainable restoration planning**

Raissa Fon Na-ah^1,2^, Nadine Ndabeh Ngwa^1^, [Liliane Ngoune Tandzi](https://sciprofiles.com/profile/1364517)^3^, Eric Ngansop Tchatchouang^1,4^, Dessireé P. Zerpa Catanho^5^*,* Emmanuel Youmbi^1^, Libert Brice Tonfack^1*^

^1^Department of Plant Biology, Faculty of Science, University of Yaounde I, P.O. Box 812, Yaounde, Cameroon.

^2^Department of Plant Biology, University of Illinois at Urbana-Champaign, Urbana, IL, USA.

^3^College of Technology, University of Bamenda, P.O. Box 39, Bambili, Cameroon.

^4^Cameroon National Herbarium, P.O. Box 1601, Cameroon.

^5^Institute of Sustainability, Energy and Environment, University of Illinois at Urbana-Champaign, 61801, Urbana, IL, USA


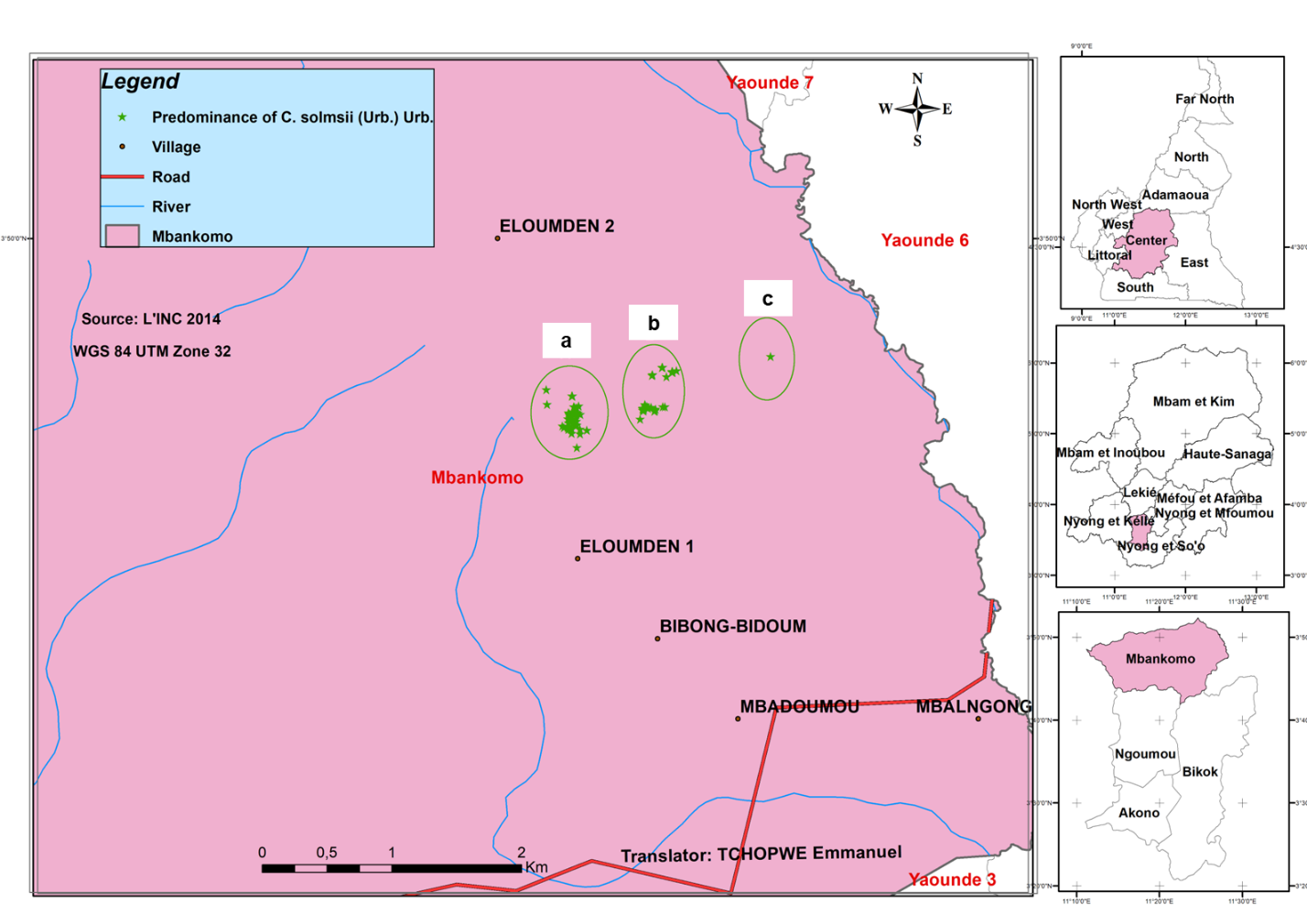


**Fig. S1.** Distribution of *C. solmsii* *(Urb.) Urb* at the submontane forest of Eloumden 1 and Eloumden 2 located in the Center Region of Cameroon. (a), (b), (c), represents habitat with high species density of wild individuals. The National and Divisional boundaries were obtained from the Geoportal of Cameroon National Herbarium, BP 1601, Yaoundé with a WGS 82 UTM Zone 32 format.


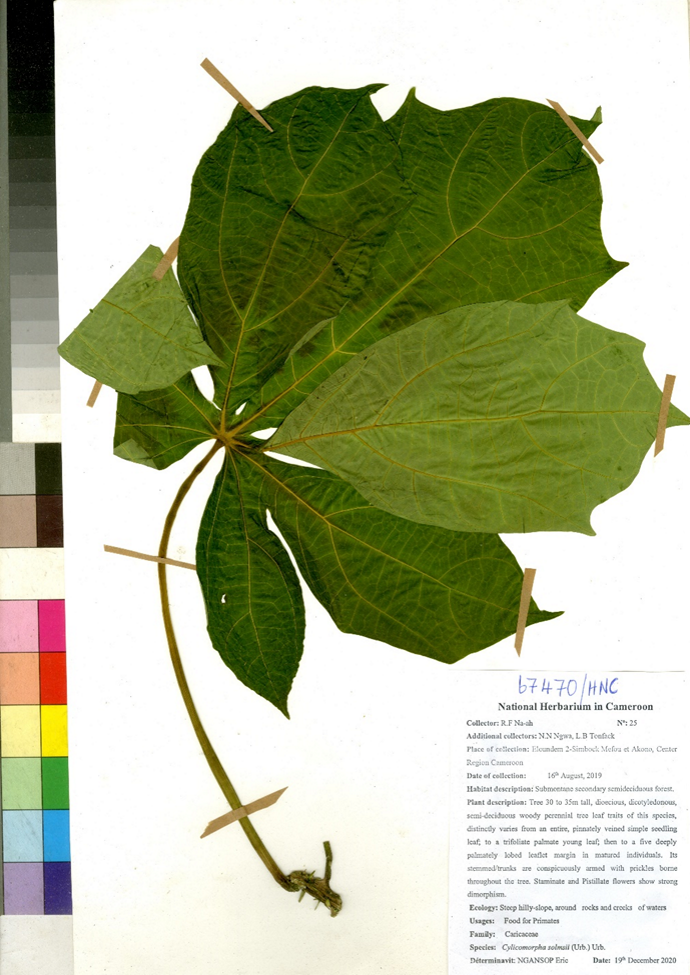


**Fig. S2.** Voucher specimens of pressed leaf sample of *C. solmsii* at the National herbarium of Yaounde, Cameroon


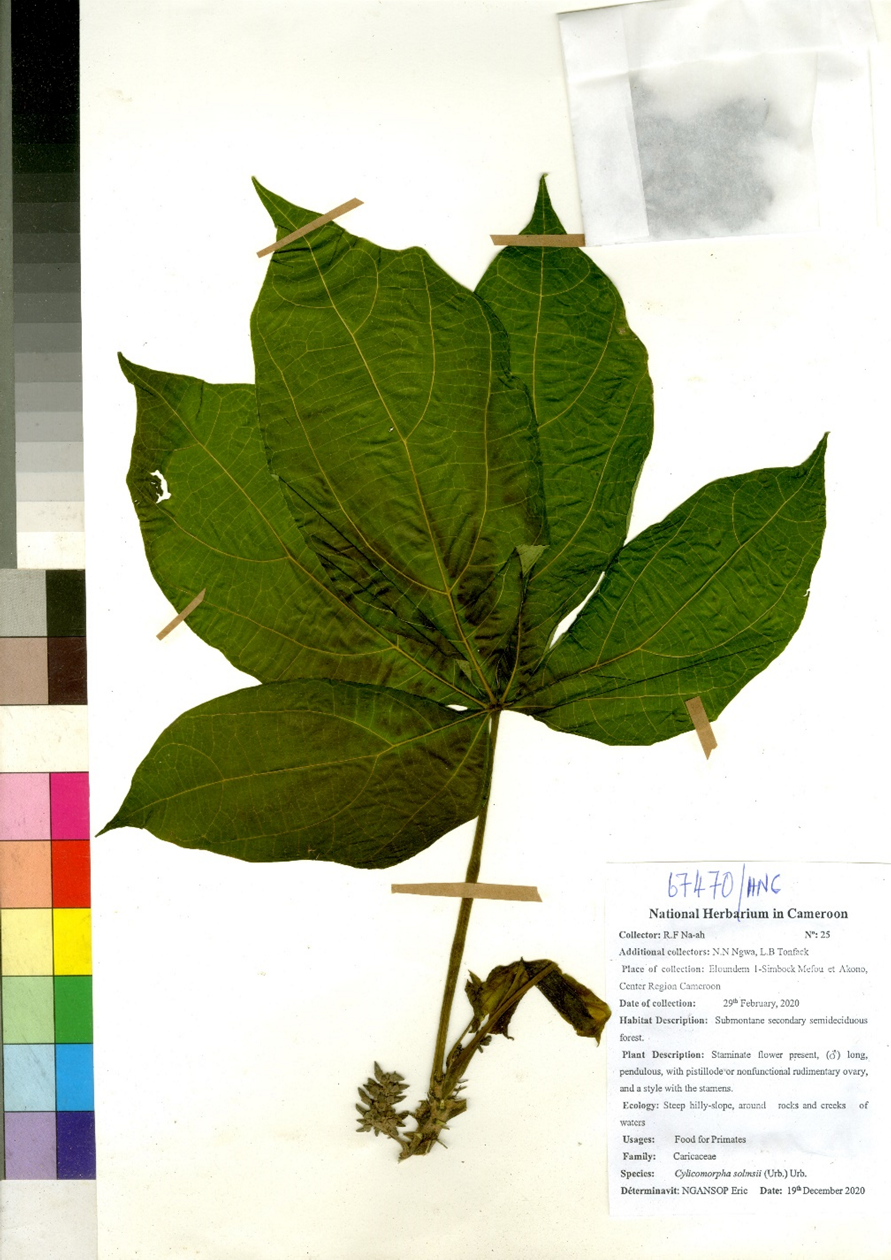


**Fig. S3.** Voucher specimens of pressed leaf and reproductive structure of *C. solmsii* at the National herbarium of Yaounde, Cameroon

**Table S1.** Textual properties and physicochemical components of soil sampled for seed gemination testing of *C. solmsii* from Eloumden I, Eloumden II and University of Yaoundé I (Yaounde I). *All physicochemical components of soil were measured in ppm, including: soluble sulfur (S), Phosphorus (P), Calcium (Ca), Magnesium (Mg), Potassium (K), Sodium (Na), Boron (B), Iron (Fe), Manganese (Mn), Copper (Cu), Zinc (Zn), Aluminum (Al), Nitrate (NO_3_^-^) and ammonium nitrate (NH_4_NO_3_), Bay 1 phosphorus.


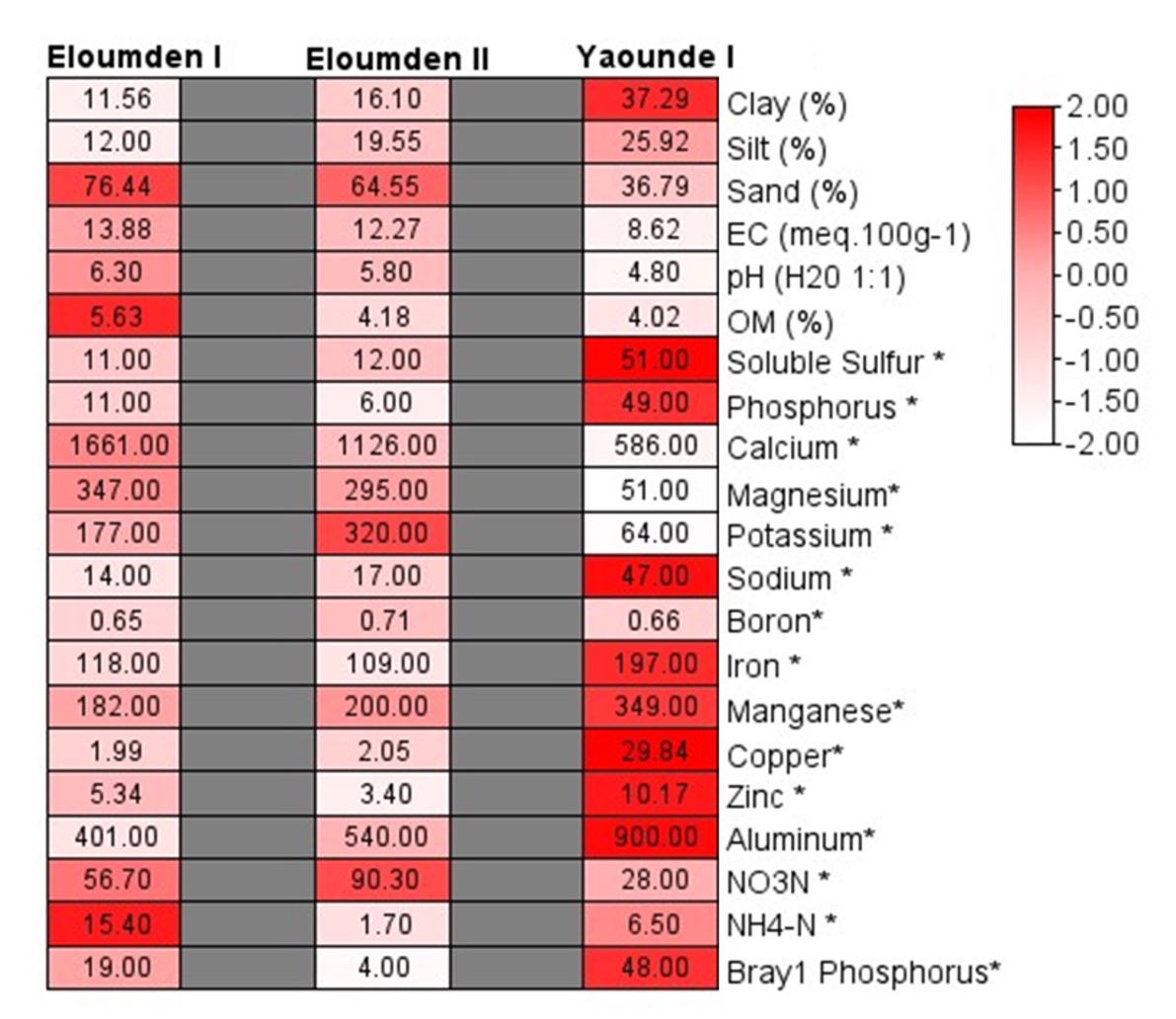


|  | **Table S2 \| Plant and soil sampling sites for germination testing of C. *solmsii*** | | | |
| --- | --- | --- | --- | --- |
| **Location** | **Sample** | **Geographical Coordinates** | | |
|  |  | **Altitude (m)** | **Longitude** | **Latitude** |
| **Eloumden I** | Plant and soil | 754 | N03°49'11.0'' | E011°26'23.9'' |
|  | Plant and soil | 768 | N03°49'13.5'' | E011°26'19.8'' |
|  | Plant and soil | 773 | N03°49'13.4'' | E011°26'19.5'' |
|  | Plant and soil | 806 | N03°49'29.7'' | E011°26'39.5'' |
|  | Plant and soil | 822 | N03°49'29.8'' | E011°26'38.5'' |
| **Eloumden II** | Plant and soil | 766 | N03°49'27.8'' | E011°26'41.2'' |
|  | Plant and soil | 774 | N03°49'25.9'' | E011°26'38.0'' |
|  | Plant and soil | 780 | N03°49'14.2'' | E011°2618.9'' |
|  | Plant and soil | 783 | N03°49'16.4'' | E011°26'19.0'' |
|  | Plant and soil | 794 | N03°49'16.3'' | E011°26'20.0'' |
| **University of Yaoundé I** | Soil | 743 | N03°51'24.0'' | E011°29'57.5'' |
|  | Soil | 737 | N03°51'24.2'' | E011°29'59.9'' |
|  | Soil | 741 | N03°51'24.3'' | E011°29'58.6'' |
|  | Soil | 744 | N03°51'23.8'' | E011°29'57.5'' |
|  | Soil | 768 | N03°51'25.3'' | E011°29'58.2'' |

| **Table S3 \| Analysis of Variance of Germination Percentages on Growth Media** | | | | | |
| --- | --- | --- | --- | --- | --- |
| ᶲ | Df | Sum Sq | Mean Sq | F value | *P(>F)* |
| Growth Media | 1 | 282 | 282 | 4.082 | 0.0899 |
| Residuals | 6 | 414.6 | 69.1 |  |  |
| Rowᶲ represent *Df =* Degrees of Freedom; Sum Sq = Sum of Squares; Mean Sq = Mean Sum of Squares; F value = F-statistic; significant levels between groups, *P* (*>F*) | | | | | |

| **Table S4 \| Growth media germination percentage mean comparison** | | | | | | | | |
| --- | --- | --- | --- | --- | --- | --- | --- | --- |
| Growth Media | ᶲ GP (%) | SD | R | SE | SEM | Min | Max | Sig |
| Petri-dish | 29.688 | 6.24 | 4 | 3.12 | 4.156289 | 25 | 38.75 | a |
| Soil | 17.813 | 9.963 | 4 | 4.982 | 4.156289 | 7.25 | 29.75 | a |
| Row ᶲ represent GP (%), Mean germination percentage value for each group; SD, standard deviation within each group; SEM, standard error of the mean; Min, minimum value within each group; Max, maximum value within each group; Sig, level of significance | | | | | | | | |

| **Table S5 \| Germination indices showing the Germination: Rate, Percentages and Mean Time of *C. solmsii*** | Germination Indices | Coefficient of variability | 60.88 | 63.1 | 73.86 | 58.01 | 64.73 | 62.86 | 66.61 | 54.43 | ⁎ T0: Freshly harvested un-scarified seeds; T1: Scarified-Fresh-Seeds; T2: Dried Scarified seeds; T3: Freeze Scarified seeds. S0: Sandy-loam¬–S0, soil from El-I and El-II mixed in a 1:1 |
| --- | --- | --- | --- | --- | --- | --- | --- | --- | --- | --- | --- |
|  |  | Seedlings Dry Weight | 17.71 | 13 | 17.44 | 13.89 | 16.2 | 15.54 | 11.86 | 12.93 |  |
|  |  | Seedling Vigor Index | 313.81 | 169.09 | 304.31 | 193.01 | 262.55 | 241.51 | 140.63 | 167.22 |  |
|  |  | Synchrony | 0 | 0 | 0 | 0.01 | 0.02 | 0.02 | 0.03 | 0.03 |  |
|  |  | Speed | 3.44 | 4.85 | 4.23 | 4.18 | 3.99 | 4.04 | 5.62 | 4.21 |  |
|  |  | Mean Rate | 0.03 | 0.05 | 0.04 | 0.04 | 0.04 | 0.04 | 0.06 | 0.04 |  |
|  |  | Mean Time | 29.1 | 20.61 | 23.62 | 23.95 | 25.03 | 24.75 | 17.8 | 23.76 |  |
|  |  | Percentage | 38.75 | 28.75 | 26.25 | 25 | 29.75 | 7.25 | 21.75 | 12.5 |  |
|  |  | Rate (%) | 31 | 23 | 21 | 20 | 119 | 29 | 87 | 50 |  |
|  |  | seeds sown | 80 | 80 | 80 | 80 | 400 | 400 | 400 | 400 |  |
|  | Treatment | Treatment | T0 | T1 | T2 | T3 | S0 | S1 | S2 | S3 |  |
|  |  | Growth Medium | Petri-dish | Petri-dish | Petri-dish | Petri-dish | Soil | Soil | Soil | Soil |  |

| **Table S6 \| Petri-dishes Mean Cumulative Germination Percentage comparison** | | | | | | | | |
| --- | --- | --- | --- | --- | --- | --- | --- | --- |
| Growth Media | ᶲMean | SD | R | SE | SEM | Min | Max | Sig |
| T0 | 31 | 0 | 1 | 0 | 0.0323 | 1 | 1 | a |
| T1 | 22 | 0.213 | 1.05 | 0.046 | 0.0384 | 1 | 2 | a |
| T2 | 20 | 0.224 | 1.05 | 0.05 | 0.0403 | 1 | 2 | a |
| T3 | 19 | 0.229 | 1.05 | 0.053 | 0.0413 | 1 | 2 | a |
| ᶲ represents rows with: SD = Standard deviation within each group; SEM = Standard error of the mean; Min = minimum value within each group; Max = maximum value within each group; Sig = level of significance.  Column: T0 = Freshly harvested un-scarified seeds; T1 = Scarified-Fresh-Seeds; T2 = Dried Scarified seeds; T3 = Freeze Scarified seeds.     \| **Table S7 \| Soil Mean Cumulative Germination Percentage comparison** \| \| \| \| \| \| \| \| \| \| --- \| --- \| --- \| --- \| --- \| --- \| --- \| --- \| --- \| \| Growth Media \| ᶲMean \| SD \| R \| SE \| SEM \| Min \| Max \| Sig \| \| S0 \| 44 \| 1.59 \| 2.7 \| 0.24 \| 0.191 \| 1 \| 9 \| a \| \| S1 \| 22 \| 0.568 \| 1.32 \| 0.121 \| 0.271 \| 1 \| 3 \| c \| \| S2 \| 35 \| 1.36 \| 2.49 \| 0.23 \| 0.215 \| 1 \| 6 \| ab \| \| S3 \| 27 \| 0.907 \| 1.85 \| 0.175 \| 0.244 \| 1 \| 5 \| bc \| \| Rowᶲ represent: SD = Standard deviation within each group; SEM = Standard error of the mean; Min = minimum value within each group; Max = maximum value within each group; Sig = level of significance.  Column: S0 = Soil from El-I and El-II mixed in a 1:1 ratio; S1= Parent soil from UY1; S2: Eloumden + Sand, mixed in a 3:1 ratio; S3; UY1+ Sand mixed in a 3:1 ratio \| \| \| \| \| \| \| \| \| | | | | | | | | |

| **Table S8 \| Analysis of Variance of Mean Germination Time on Growth Media** | | | | | |
| --- | --- | --- | --- | --- | --- |
| ᶲ | Df | Sum Sq | Mean Sq | F value | *P*(*> F*) |
| Growth Media | 1 | 4.43 | 4.429 | 0.37 | 0.565 |
| Residuals | 6 | 71.78 | 11.964 |  |  |
| Rowᶲ represent: Df = Degrees of Freedom; Sum Sq = Sum of Squares; Mean Sq = Mean Sum of Squares; F value = F-statistic; significant levels between groups | | | | | |

| **Table S9 \| Growth Media Mean Germination Time mean comparison** | | | | | | | | |
| --- | --- | --- | --- | --- | --- | --- | --- | --- |
| ᶲGrowth Media | ᶲ GP(%) | SD | R | SE | SEM | Min | Max | Sig |
| Petri-dish | 24.319 | 3.522 | 4 | 1.761 | 1.729437 | 20.61 | 29.09677 | a |
| Soil | 22.831 | 3.394 | 4 | 1.697 | 1.729437 | 17.8 | 25.03361 | a |
| ᶲ represent: GP (%) = Mean germination percentage value for each group; SD = Standard deviation within each group; SEM = Standard error of the mean; Min = minimum value within each group; Max = maximum value within each group; Sig = level of significance | | | | | | | | |

| **Table S10 \| Intraclass Correlation Coefficients (ICCs): Test of Independent Assumption** | | | | | | | | |
| --- | --- | --- | --- | --- | --- | --- | --- | --- |
|  | Type | ICC | F | df1 | df2 | *P* | Lower  bound | Upper  bound |
| Single rater’s absolute | ICC1 | -0.129 | 0.43 | 19 | 80 | 0.98 | -0.18 | -0.01 |
| Single random raters | ICC2 | 0.041 | 1.99 | 19 | 76 | 0.019 | -0.01 | 0.14 |
| Single fixed raters | ICC3 | 0.165 | 1.99 | 19 | 76 | 0.019 | 0.01 | 0.41 |
| Average absolute rater | ICC1k | -1.322 | 0.43 | 19 | 80 | 0.98 | -3.42 | -0.04 |
| Average random raters | ICC2k | 0.176 | 1.99 | 19 | 76 | 0.019 | -0.04 | 0.46 |
| Average fixed raters | ICC3k | 0.497 | 1.99 | 19 | 76 | 0.019 | 0.04 | 0.78 |

| **Table S11 \| Residual Standard Errors of Soil treatment on Seedling Growth of *C. solmsii* from MANOVA analysis** | | | | | | |
| --- | --- | --- | --- | --- | --- | --- |
|  | Treatment | Residuals |  |  |  |  |
| Plant height | 4195.402 | 594.948 |  |  |  |  |
| Stem diameter | 85.8 | 124.4 |  |  |  |  |
| Leaf number | 1.75 | 10.8 |  |  |  |  |
| Petiole length | 82.942 | 53.744 |  |  |  |  |
| Chlorophyll content | 138.044 | 624.224 |  |  |  |  |
| Leaf area | 80.962 | 686.156 |  |  |  |  |
|  | Df | Pillai | Approx F | Num Dfᶲ | Den Dfᶲ | Pr(>F) |
| Treatment | 3 | 1.3148 | 1.6904 | 18 | 39 | 0.08439 |
| Residuals* | 16 |  |  |  |  |  |
| *Residual standard errors: 6.10, 2.79, 0.82, 1.83, 6.25, 6.55, represent variability not explained by the model,  ᶲ Respectively denotes Num Df = Numerator Degrees of Freedom and Den Df = Denominator Degrees of Freedom | | | | | | |

| **Table S12 \| One-Way ANOVA showing the influence Of Soil Treatment on the Seedling Growth of *C. solmsii*** | | | | | | |
| --- | --- | --- | --- | --- | --- | --- |
| **Soil Treatment** | **Plant Growth Parameter** | | |  |  |  |
|  | Df | SSᶲ | MSᶲ | F-Value | *P*(>F) | Significance level |
|  | **Plant Height** | | | | | |
| Soil Treatment | 3 | 4195.4 | 1398.47 | 37.609 | 1.78E-07 | *** |
| Residuals | 16 | 594.9 | 37.18 |  |  |  |
| **Stem Diameter** | | | | | | |
| Soil Treatment | 3 | 85.8 | 28.6 | 3.6785 | 0.034 | * |
| Residuals | 16 | 124.4 | 7.775 |  |  |  |
| **Leaf Number** | | | | | | |
| Soil Treatment | 3 | 1.75 | 0.58333 | 0.8642 | 0.48 |  |
| Residuals | 16 | 10.8 | 0.675 |  |  |  |
| **Petiole Length** | | | | | | |
| Soil Treatment | 3 | 82.942 | 27.647 | 8.2308 | 0.0015 | ** |
| Residuals | 16 | 53.744 | 3.359 |  |  |  |
| **Leaf Area** | | | | | | |
| Soil Treatment | 3 | 80.96 | 26.987 | 0.6293 | 0.61 |  |
| Residuals | 16 | 686.16 | 42.885 |  |  |  |
| **Chlorophyll Content** | | | | | |  |
| Soil Treatment | 3 | 138.04 | 46.015 | 1.1794 | 0.3487 |  |
| Residuals | 16 | 624.22 | 39.014 |  |  |  |
| ᶲ respectively represents sum of squares (SS) and mean squares (MS) | | | | | | |
|  | | | | | | |

| **Table S13 \| Tukey's multiple pairwise comparisons showing the mean difference soil group at each level** | | | | | |
| --- | --- | --- | --- | --- | --- |
| **ᶲContrast** | **Estimate** | **SE** | **Df** | **T ratio** | ***P* value** |
| S0-S1 | 6.223 | 1.09 | 16 | 5.71 | 0.0002 |
| S0-S2 | 0.74 | 1.09 | 16 | 0.679 | 0.9036 |
| S0-S2 | 6.94 | 1.09 | 16 | 6.367 | 0.0001 |
| S1-S3 | -5.483 | 1.09 | 16 | -5.031 | 0.0006 |
| S1-S2 | 0.716 | 1.09 | 16 | 0.657 | 0.9115 |
| S2-S3 | 6.2 | 1.09 | 16 | 5.688 | 0.0002 |
| ᶲThe row names contrast between two groups: estimated difference between two groups. Df = degrees of freedom; t-statistics and p-value associated with the contrast | | | | | |
|  | | | | | |
|  | | | | | |

| **Table S14 \| Predictive Linear Model showing the effect of each soil treatment on an individual plant growth parameter** | | | | | | |
| --- | --- | --- | --- | --- | --- | --- |
| **ᶲSoil Treatment** | **Plant Growth Parameter** | | | | | |
| **Plant Height** | | | | | | |
|  | Estimate | Std. Error | t value | *P* (>\|t\|) |  |  |
| Intercept | 52 | 2.727 | 19.068 | 1.99E-12 | *** |  |
| Treatment S1 | -33.18 | 3.857 | -8.603 | 2.13E-07 | *** |  |
| Treatment S2 | -3.8 | 3.857 | -0.985 | 0.339 |  |  |
| TreatmentS3 | -27.8 | 3.857 | -7.208 | 2.09E-06 | *** |  |
|  | RSE | Df | MRS | ARS | F-Stat | p-value |
|  | 6.098 | 16 | 0.8758 | 0.8525 | 37.61 | 1.78E-07 |
|  | Min | 1Q | Median | 3Q | Max |  |
|  | -11.7 | -3.175 | -1.2 | 4.675 | 8.8 |  |
| **Stem Diameter** | | | | | | |
| Intercept | 13.6 | 1.247 | 10.906 | 8.11E-09 | *** |  |
| Treatment S1 | -3.6 | 1.764 | -2.041 | 0.0581 | . |  |
| Treatment S2 | -0.6 | 1.764 | -0.34 | 0.7381 |  |  |
| Treatment S3 | -5 | 1.764 | -2.835 | 0.0119 | * |  |
|  | RSE | Df | MRS | ARS | F-Stat | p-value |
|  | 2.788 | 16 | 0.2972 |  | 3.678 | 0.03452 |
|  | Min | 1Q | Median | 3Q | Max |  |
|  | -4.6 | -1.95 | 0.4 | 1.55 | 4 |  |
| **Leaf Number** | | | | | | |
| Intercept | 3 | 0.3674 | 8.165 | 4.25E-07 | *** |  |
| Treatment S1 | 0.6 | 0.5196 | 1.155 | 0.265 |  |  |
| Treatment S2 | -0.2 | 0.5196 | -0.385 | 0.705 |  |  |
| Treatment S3 | 0.2 | 0.5196 | 0.385 | 0.705 |  |  |
|  | RSE | Df | MRS | ARS | F-Stat | p-value |
|  | 0.8216 | 16 | 0.1394 | -0.0219 | 0.8642 | 0.4798 |
|  | Min | 1Q | Median | 3Q | Max |  |
|  | -1.6 | -0.2 | 0 | 0.2 | 2.4 |  |
| **Petiole Length** | | | | | | |
| Intercept | 10.06 | 0.8196 | 12.274 | 1.48E-09 | *** |  |
| Treatment S1 | -4.48 | 1.1591 | -3.865 | 0.00137 | ** |  |
| Treatment S2 | -0.4 | 1.1591 | -0.345 | 0.73453 |  |  |
| Treatment S3 | -4.02 | 1.1591 | -3.468 | 0.00317 | ** |  |
|  | RSE | Df | MRS | ARS | F-Stat | p-value |
|  | 1.833 | 16 | 0.6068 | 0.5331 | 8.231 | 0.001538 |
|  | Min | 1Q | Median | 3Q | Max |  |
|  | -3.28 | -0.875 | 0.03 | 1.01 | 2.84 |  |
| **Leaf Area** | | | | | | |
| Intercept | 33.08 | 2.929 | 11.295 | 4.92E-09 | *** |  |
| Treatment S1 | 4.4 | 4.142 | 1.062 | 0.304 |  |  |
| Treatment S2 | -0.86 | 4.142 | -0.208 | 0.838 |  |  |
| Treatment S3 | 0.58 | 4.142 | 0.14 | 0.89 |  |  |
|  | RSE | Df | MRS | ARS | F-Stat | p-value |
|  | 6.549 | 16 | 0.1055 | -0.0622 | 0.6293 | 0.6066 |
|  | Min | 1Q | Median | 3Q | Max |  |
|  | -10.46 | -4.885 | 0.07 | 4.285 | 11.54 |  |
| **Chlorophyll Content** | | | | | | |
| Intercept | 28.3 | 2.793 | 10.131 | 2.29E-08 | *** |  |
| Treatment S1 | -1.08 | 3.95 | -0.273 | 0.788 |  |  |
| Treatment S2 | 1.42 | 3.95 | 0.359 | 0.724 |  |  |
| Treatment S3 | -5.598 | 3.95 | -1.417 | 0.176 |  |  |
|  | RSE | Df | MRS | ARS | F-Stat | p-value |
|  | 6.246 | 16 | 0.1811 | 0.02755 | 1.179 | 0.3487 |
|  | Min | 1Q | Median | 3Q | Max |  |
|  | -10.46 | -4.885 | 0.07 | 4.285 | 11.54 |  |
|  | -20.592 | -1.495 | 0.49 | 2.449 | 8.398 |  |
| ᶲIntercept =S0; S1, S2, S3 = each level of soil treatment; 1 = is row representing RSE = Residual standard error, Df = Degrees of Freedom; MRS = Multiple R-squared; ARS = Adjusted R-squared; F-Stat = F-statistic | | | | | | |
|  | | | | | | |

| **Table S15 \| Analysis of Statistical significance of the Linear Model** | | | | | | |  |
| --- | --- | --- | --- | --- | --- | --- | --- |
|  | Df | Pillai | Approx F | Num Df ᶲ | Den Df ᶲ | Pr (>F) | Significance |
| Intercept | 1 | 0.99521 | 380.97 | 6 | 11 | 4.22E-12 | *** |
| Treatment | 3 | 1.31479 | 1.69 | 18 | 39 | 0.08439 | . |
| Residuals | 16 |  |  |  |  |  |  |
| ᶲRow indicates Df = Degrees of Freedom; num Df (Numerator Degrees of Freedom); Num Df = Numerator Degrees of Freedom and Den Df = Denominator Degrees of Freedom | | | | | | | |

| **Table S16 \|** Characterization of vegetative morphological trait descriptors in *C. solmsii* | | |
| --- | --- | --- |
| **Standard scored morphological descriptor*** | *C. solmsii* | |
|  | Young ^a^ | Matured ^b^ |
| **Leaf/Leaflet** |  |  |
| **Type:** Simple (1), Compound (2) | 1 | 2 |
| **Margin:** entire (1), palmately lobed (2), pinnately lobed (3) | 1 | 2 |
| **Attachment:** petiolate (1), sessile (2), sheathing (3), decurrent (4) | 1 | 1 |
| **Phyllotaxy:** alternate (1), opposite (2), whorled (3), spiral (4) | 1 | 1 |
| **Venation:** uninervous (1), dichotomous (2), parallel (3) , pinnately netted/pinnate-veined (4), palmately veined/palmate-netted (5) | 4 | 5 |
| **Surface:** glabrous (1), tomentose (2), pubescent (3), pilose (4) | 1 | 1 |
| **Stipule :** exstipulate (0), stipulate (1) | 0 | 0 |
| **lamina Shape:** ovate (1), elliptic (2), lanceolate (3), cordate (4), oblong (5), sagittate (6) | 1 | 2:3/2:1 |
| **Apex/ base Shape of :** acuminate (1), acute (2), rounded (3), cordate (4), oblique (5), sagittate (6), hastate (7), truncate(8) | 1/4 | 1/4 |
| **lobes Number of :** zero (0), one (1), two (2), three (3), four (4), five (5) | 0–3 | 5 |
| **Color:** light green (1), dark green (2), reddish green (3), red (4) | 1, 3, 4 | 1, 4 |
| **Vein color:** green (1), reddish green (2), red (3) | 2, 3 | 2, 1 |
| **Petiole color:** green (1), reddish-green (2), greenish-red (3), red (4) | 4, 2 | 3, 1 |
| **Leaf Size: Length;** Av*: 9.1m, Min*: 6.0cm; Max*: 13.5cm, **Diameter;** Av: 9.73mm, Min: 5.1mm, Max: 13.5 mm | | |
| **Petiole : Length;** Av: 28.59cm, Min: 14.5cm; Max: 55.0cm, **Diameter;** Av: 5.24mm, Min: 3.0 mm, Max: 8.0 mm | | |
| **Stem/Trunk** |  |  |
| **Type:** acaulescent (1), caulescent (2), arborescent (3), deliquescent (4) | 3 | 3 |
| **Texture:** herbaceous (1), woody (2), chartaceous (3) | 1 | 2 |
| **Branching pattern:** dichotomous (1), pseduomonopodial (2), monopodial (3), sympodial (4) | 3 | 3 |
| **Growth patterns:** Indeterminate (1), determinate (2) | 1 | 1 |
| **Defense barrrier :** eepidermis (1), periderm (2), cork (3), bark (4) | 1 | 4 |
| **Bark/epidermal** configuration**:** exfoliating (1), fissured (2), plated (2), shreddy (3), smooth (4) | 4 | 4 |
| **Scars:** carpel scar (1), inflorescence scar (2), leaf scar (3), perianth scar (4), stamen scar (5), stipule scar (6), vein scar (7) | 3 | 3 |
| **Epidermal excrescence:** thorns (1), spines (2), prickles (3) | 3 | 3 |
| **Stem/bark color:** brown (1), purplish red (2), gold (3), light brown (4), dark brown (5), green-yellowish (6), golden (8), gray (9) orange (10) | 2, 8, 4, 9, 5 | 10, 6, 8, 4, 9, 5 |
| **Exudate type:** resins (1), latex (2), gums (3), phenolics (4) | 4 | 4 |
| a Young C. solmsii includes plants less than or equal to three months old.  b Matured C. solmsii includes plants/tree less than or equal to six months old from the wild. Av=Average; Min=Minimum; Max= Maximum | | |

| **Table S17 \|** Reproductive trait descriptors of *C. solmsii* staminate and pistillate flowers | | | |
| --- | --- | --- | --- |
| **Standard scored morphological descriptor *** | Abbr* | *C. solmsii* | |
|  |  | Immature | Mature |
| **Flower** | F |  |  |
| **Classification:** monoecious (1), dioecious (2) |  | / | 2 |
| **Sex:** bisexual - ⚥ (1), unisexual [male flower - **♂,** Female flower - ♀] (2), polygamous (3) | ⚥/♂/ ♀ | ♂ & ♀ | ♂ & ♀ |
| **Bracteoles:** ebracteoles (0), bracteoles (1) | Ebr/ Br | 0 | 0 |
| **Development:** solitary flowers (1) inflorescence (2) |  | 2 | 2 |
| **Attachment:** pedicellate (1), sessile (2), subsessile (3) |  | 1 | 1 |
| **Cycly:** complete (1), incomplete (2) |  | 2 | 2 |
| **Symmetry:** actinomorphic (1), zygomorphic (2) | ⊕/ % | 1(♂), 1 (♀) | 1(♂), 1 (♀) |
| **Inflorescence** |  |  |  |
| **Bract/Bractlet:** ebracteate (0), bracteate (1) | Ebr/ Br | 0 | 0 |
| **Type:** cymose (1), simple racemose (2), compound racemose or panicle (3) |  | 3 | 3 |
| **Development:** determinate (1), indeterminate (2) |  | 2 | 2 |
| **Position:** axillary (1), terminal (2), cauliflorous (3) |  | 1 | 1 |
| **Perianth** | P |  |  |
| **Parts:** Calyx-K (1), Corolla – C (2), Tepals – 3 (P) | K, C, P | 1, 2 | 1, 2 |
| **Arrangement:** spiral (1), whorled (2) |  | 2 | 2 |
| **Cycly:** achlamydeous (0), monochlamydeous (1), dichlamydeous (2) [homochlamydeous (a), heterochlamydeous (b)] |  | 2b | 2b |
| **Merosity:** isomerous (1), anisomerous (2) |  | 2 | 2 |
| **Fusion:** free (1) [ polysepalous (a), polypetalous (b), polytepalous (c)], fused (2) [gamosepalous (a), gamopetalous (2), gamotepalous (c)] |  | 2 | 1b, 2a |
| **Sepals duration:** caducous (1), deciduous (2), persistant (3) |  | 2 | 2 |
| **Corolla shape:** rotate (1), campanulate (2), urceolate (3) infundibular (4), salverform (5), tubular (6), chalice (7) |  | 6(♂, ♀) | 5(♂), 7(♀) |
| **Aestivation:** valvate (1), twisted (2), imbricate (3), quincuncial (4), vexillary (5) |  | 2 (♂), 1 (♀) | |
| **Corolla colour:** white (1), cream-white(2), deep yellow (3), yellowish green (4), dark green (5), light green (6), yellowish green and red shades (7) |  | 4,6 (♂), 6,7 (♀) | 1, 2 (♂), 4,6 (♀) |
| **Androecium or Stamen** | A |  |  |
| **Type:** laminar (1), filamentous (2) |  | / | 2 |
| **Arrangement:** whorled (1), spiral (2), didynamous (3), tetradynamous (4) |  | / | 1 |
| **Cycly:** uniseriate (1), biseriate (2) |  | / | 2 |
| **Position:** antisepalous (1), alternipetalous (2) diplostemonous (3) obdiplostemonous (4) |  | / | 2 |
| **Fusion:** distict (1), connate (2) adnate (3), [epipetalous (a), epitepalous (b), episepalous (c)] | A _()_ | 2 | 3, 2 (a) |
| **Stamen Shape:** present / oblanceoloid, filiform |  |  |  |
| **Stamen Colour:** |  |  |  |
| **Male Floral Formular :** **⊕♂ K _(1)_C_5_****^⤺^A _(5) +(5)_** | | | |
| **Gynoecium** | G |  |  |
| **Type: simple (1), compound (2)** |  | 2 | 2 |
| **Fusion:** syncarpous (1), apocarpous (2) | G _()_ | 1 | 1 |
| **Ovary Position:** inferior (1), superior (2) |  | 2 | 2 |
| **Position of Perianth:** hypogynous (1), perigynous (2), epigynous (3) |  | 1 | 1 |
| **Carpel/locule Number:** 1, 2, 3, 4, 5, 10 |  | 5 | 5 |
| **Placentation:** axile (1), parietal (2), basal (3) |  | 2 | 2 |
| **Stigma Shape:** capitate (1), plumose (2), discoid (3), dumb-bell (4),) funnel (6), |  | / | 6 |
| **Ovury Shape:** |  |  | |
| **Female Floral Formular:** **⊕ ♀K _(1)_C_5_G _(5)_** | | | |
| *Note.* * See Table 1 for sources Standard scored morphological trait descriptor. ***** Abbreviation of floral symbols from (Simpson 2013). Numbers and paranthese below a floral whorl respresents merosity and fusion of whorl or | | | |

| **Table S18 \|** Fruits, Seeds, Seedlings, Root architecture of *C. solmsii* | | |
| --- | --- | --- |
| **Standard scored morphological descriptor *** | *C. solmsii* | |
|  | Immature | Mature |
|  |  |  |
| **Fruit** |  |  |
| **Type:** simple [ Fleshy-Drupe, Berry; Dry ] (1), aggregate (2), composite (3) | 1 | 1 |
| **Postion of Pedicel: s**ymetrical (1), asymmetrical (2) | 1 | 1 |
| **Stigma scar:** absent (0), present (1) | 1 | 1 |
| **Dispersal:** anemochory (1), autochory (2), hydrochory (3), zoochory (4) | 4 | 4 |
| **Size : Length** Av: 4.7cm, Min: 3.3cm; Max: 5.8cm; **Diameter** Av: 28.0mm, Min: 26.0mm, Max: 36.0mm; **Weight;** Av: 22.05g ; Min: 19.76g Max: 36.777g | | |
| **Shape:** ovate (1), elliptic (2), lanceolate (3), obovate (4) | 1, 2 | 1, 2 |
| **Color:** Green (1), light green (2), yellowish-green (3) dark green (4) | 2 | 3 |
| **Seed** |  |  |
| **Type:** orthodox (1), recalcitrant (2), intermediate (3) | 3 | 3 |
| **Endosperm Type:** endospermous (1), nonendospermous (2), cotylespermous (3) | 1 | 1 |
| **Germination Type:** hypogeous or cryptocotylar (1) or epigeous or phanerocotylar (2) | N/A | 2 |
| **Dispersal:** anemochory (1), autochory (2), hydrochory (3), zoochory (4) | 3 | 3 |
| **Size: Length;** Av: 0.6cm, Min: 0.4cm; Max: 0.7cm, **Diameter;** Av: 3.5mm, Min: 3.0mm, Max: 4mm **Weight;** Av: 0.0126g ; Min: 0.01g Max: 0.039g | | |
| **Number of seed** **per fruit:** Av: 202, Min:14, Max:318 | | |
| **Color:** gold (1), striped golden-brown (2), striped whitish-brown (3), brown (4) | 3 | 2 |
| Plant |  |  |
| **Habit:** Herb (1), shrub (2), tree (3), vine (4); Single trunk (a), Multiple trunk (b) | 1/a | 3/a |
| **Duration: Plant;** annuals (1), biennials (2), perennials (3;  **Leaf;** evergreen (a), deciduous (b), semi-deciduous (c) | 3/a | 3/c |

| **Table S19 \|** Morphometric measures of *C. solmsii* | | | | | |
| --- | --- | --- | --- | --- | --- |
| **Quantitatively measurements** | **Sample number** | **Mean** | **Min** | **Max** | **Range** |
| Leaf length (cm) | 50 | 9.46 ± 0.2 | 6.1 | 13.5 | 7.4 |
| Leaf diameter (mm) | 50 | 9.73 ± 0.15 | 5.1 | 13.5 | 2.4 |
| Petiole length (cm) | 50 | 28.59 ±7.95 | 14.5 | 55 | 40.5 |
| Petiole diameter (mm) | 50 | 5.24 ± 1.00 | 3 | 8 | 5 |
| Fruit weight (g) | 50 | 22.05 ± 4.86 | 9.18 | 36.78 | 27.6 |
| Fruit length (cm) | 50 | 4.738 ± 0.590 | 3.3 | 5.8 | 2.5 |
| Fruit diameter (mm) | 50 | 28.5 ± 4.88 | 26 | 36 | 33 |
| Length of fruit peduncle(cm) | 50 | 6.00 ± 0.051 | 3 | 12 | 9 |
| Seed length (cm) | 50 | 0.60 ± 0.05 | 0.5 | 0.7 | 0.2 |
| Seed diameter (mm) | 50 | 3.00 ± 0.28 | 2 | 4 | 2 |
| Seed number (count) | 50 | 202.47 ± 74.69 | 14 | 318 | 304 |
| Seed fresh weight (g) | 5 | 0.45 ± 0.01 | 0.23 | 0.59 | 0.05 |
| Seed dry weight (g) | 5 | 0.01 ± 0.002 | 0.001 | 0.04 | 0.04 |
| Seed dry weight of 100 seeds (g) | 10 | 2.90 ± 1.21 | 0.44 | 5.05 | 4.61 |
